# Ninth International Medical Congress

**Published:** 1887-09

**Authors:** 


SECOND DAY—AFTERNOON SESSION.

Professor W. H. Wathen, of Louisville, Kentucky, read a paper on the subject,

RAPID DILATATION OF THE CERVIX UTERI.

The author had learned by experience and observation of the bad results from efforts to dilate the cervical canal by tents, or to enlarge oı straighten it by incisions to cure dysmenorrhœa and sterility. He wished to call attention to the more satisfactory means of rapid dilatation with the bivalve or double-bladed dilators now in use, and especially to the substitution of an instrument of his device for Goodell's modification of Ellinger's dilator. If tents were used, he preferred the tupello to any other variety, it being less apt to cause septic inflammation than the sponge, and dilated more rapidly, regularly, and better than the tangle. He referred to endometritis, pelvic hæmatocele, pelvic cellular or peritoneal inflammation, septicæmia, pyæmia, and tetanus, as complications accompanying or following the use of tents, and did not believe that any good results, apparently, were permanently obtained. He claimed that the two-bladed dilators are relatively aseptic and are easily used; complete the operation at one sitting, and that the dilatation is comparatively free from immediate or subsequent dangers. It nearly always cures the dysmenorrhœa, and often removes the causes of sterility.

He thought the results of the incision of the cervix up to the vaginal junction, or through the os internal, anteriorly, posteriorly, or bi-laterally, even more unsatisfactory than those following the use of tents. He dilates the cervix in his office, without a local or general anæsthetic, to the extent of half an inch, and allows the woman to walk or ride home a few minutes after. In dilatations of from three-quarters to one inch, he gives a hypodermic injection of morphia and atropia, then brings the patient under the influence of chloroform before operating. He urges great cleanliness, and all means to prevent septic infection. He uses three sizes of dilators, the largest the one he devised. He explained the points of supposed superiority over Goodell's.

In conclusion, he urged that the operation should not be performed if there is any pelvic inflammation or trouble in the tubes or ovaries; and never, in any case, until we are reasonably positive that the cause of the trouble is in the cervical canal.

Dr. A. Martin, of Berlin, Prussia, said that the dilatation of the uterus as an operation had undergone remarkable changes since its origination. The instruments shown by Dr. Wathen are an improvement. The great object is to open up the internal os. The degree of dilatation may be required to be less in some cases of small cervix. It is a question whether we have not been dilating in too many cases. He would not recommend that we go back to laminaria and sponge-tents.

Dr. S. H. Weeks, of Portland, Maine, claimed that every case of dysmenorrhœa was not due to mechanical obstruction of the cervix. The ovaries and tubes have much to do with it, and in his experience have been oftener at fault than the cervical canal. He was surprised at the freedom from danger reported in this operation. Professor Goodell has reported one hundred cases with no bad result. These instruments are not devoid of danger, and bad results may come.

Dr. C. R. Reed, of Middleport, Ohio, thought it quite puzzling to the young gynæcologist to tell what to do, the testimony being so conflicting. Some authors reported that there never had been any bad results in the few hundred cases that they had operated on; others had frequent fatalities. He has more hesitancy in operating now than formerly. We cannot predict what the result will be in a given case.

Dr. Goelet, of New York, N. Y., thought that the term rapid dilatation given to the operation was a misnomer. It had come into use to distinguish it from the dilatation by sponge or laminaria-tents.

Dr. Steason, of North Carolina, performed dilatation many times a year, for the past ten years. He keeps his patients in bed but two days. He has never had any fever follow, and has not used any antiseptic except soap and water. He uses a glass rod to keep the canal open much the same as does Dr. Goelet, leaving it there sometimes as long as a week. He did not believe in the superfluous use of so much antiseptic.

Professor A. Reeves Jackson, of Chicago, Illinois, objected to the operation, because it did not do any good, at least permanent good.

Dr. Hadley, of Australia, never uses sponges and laminaria in dilatation, which he reviewed in extenso.

Dr. Lawrence, of Bristol, England, had used sounds constantly for years. His patients are constantly coming back. In the unimpregnated uterus, with proper precautions, this treatment can be used but must not be left in too long. He uses gelatine-coated sponge-tents, previously saturated with carbolic acid.

Professor Wathen, in conclusion, said as to the question concerning his operating at his office, he did it without any concern, and no anæsthetic. As to Emmett's opinion, we will concede a great deal to him in gynæcology, but he does not see how Emmett can be taken as an authority in this subject, seeing that he has not performed the operation, condemns it in toto, and will have nothing to do with it. The happy medium in this, as in other cases, is the thing to follow. No one would dilate for dysmenorrhœa caused by cellulitis or inflammation. The operation can be performed to straighten the neck of the uterus, and we can shorten the neck of the uterus by dilating the cervix and without amputation.

---

### SECTION ON OPHTHALMOLOGY.

### Second Day—Morning Session.

Mr. Henry Power, of London, England, presented a paper entitled

### MICROBES IN THE DEVELOPMENT OF EYE DISEASES.

After a historical sketch of the earlier researches of Pasteur and those who followed immediately after him in the discovery of the nature and modes of propagation of microbes, and the demonstration of Huxley and others that diseases were developed by these organisms, and that they would grow and form colonies in especial situations, the reader went over the discovery of the micrococcus of syphilis and the gonococcus of ophthalmia. Chalozion, pterygium, blepharitis ciliaris, purulent conjunctivitis, kerato-iritis, optic neuritis, cerebral meningitis, may be considered to be the result of microbic development. The factors in the production of these effects may be as follows:

1. They may first penetrate into the tissues and use up the pabulum necessary for their growth.

2. They may use up the whole of the oxygen, and so deprive the tissue of a necessary element ; or

3. They may throw out what we may call ptomaines, poisonous products.

He related a case of a gentleman who, after a fatiguing walk of some hours, had a sudden accession of pain in the eye and developed an acute conjunctivitis. His opinion was that, though the tissues in a state of health were able to resist the attack of the ordinary microbes, as found in the air of cities and dense populations, that when this power of resistance was diminished by fatigue they were able to effect a lodgment and cause disease. He spoke of the experiment with a long tube, coated inside with agar agar or gelatine, through which microbic air was passed, showing that different varieties of microbe settled and formed colonies at different parts of the inclined tube, illustrating the fact that microbes were heavy enough to settle, and in different degrees.

The lessons which he proposed to draw from this were, to avoid all conditions which may lower the condition of the system, remove the patient from the densely populated parts, and in this connection, remember that it has been shown that at sea, at a certain distance from land, the air contains absolutely no microbes. Of course, in crowded ships this condition does not obtain.

Dr. Abadie, of Paris, France, made a few remarks on the absolute necessity of clean instruments, and cleanliness in regard to all accessories, in eye operations, and was followed by Dr. N. Monolescu, of Bucharest, in the same strain.

Dr. Heyl, of Philadelphia, Pennsylvania, said that it is evident that two general errors in regard to the subject have been developed. One is, that if a remedy will arrest the progress of the microbe in a gelatine solution, it will be applicable to the diseased conditions produced by that microbe, and also that any antiseptic may be used one for another. The microbe attacks the older and feebler cells in purulent conjunctivitis, and nitrate of silver is of use in destroying these old cells and leaving only the young and vigorous, that are able to resist the microbic attacks. The exacerbations occur as these young cells become older and feebler, and are again cut short by the silver application. Diphtheritic follows closely the purulent form in course and causation in this connection.

He found the reason for destructive changes in the cornea in these affections in the different application of the epithelial layer to the subjacent tissue. This was also a factor in hemeralopia, the disease being considered as an epithelial mycosis, the palpebral epithelium being lubricating while the sclero-corneal is intimately connected with the function of vision.

Hypopion keratitis is an affection of the lymph itself. There has been a solution of continuity and the infection gets into the eye. Thus also, in cataract extraction the wound lays the eye open to the approach of the microbe. In kerato-hypopion and abscess, a good result was obtained by the use of a salve of agnine having incorporated oleo-resin of cubebs. The application seemed to be quite painful, the patient sometimes being kept awake all night by it. A good application is a salve of red cinchona applied on the conjunctiva. A satisfactory remedy in kerato-hypopion is the application of iodoform to the ulcer.

Dr. B. J. Baldwin, of Montgomery, Alabama, hoped that Dr. Power would also present the surgical side of microbe infection, which is now the liveliest question in eye surgery. He wished to present the other side of the case. In cataract operations he used no protection again the invasion of microbes, except to wash the patient's eye and face. In old hospitals it may be as well to wash and use all these precautions, but in new ones simple attention to cleanliness only is necessary. Dr. Baldwin was doubtful whether any better results were obtained now than before the introduction of antisepsis in eye surgery.

Dr. Landolt, of Paris, France, was astonished to see how little attention is paid to antisepsis in eye operations, and that we know too little of bacteria in eye affections. His experience was in favor of 1 to 5,000 solution of sublimate in extractions. He saw no objection to the solution. It did not prevent union or damage anything.

Professor D. S. Reynolds, Louisville, Kentucky, remarked that he had not for a number of years had suppuration of the cornea after cataract extractions. He insisted on absolute cleanliness, and, where that could not be secured, declined to operate.

Professor P. D. Keyser, Philadelphia, Pennsylvania, remarked that the main argument in favor of antisepsis is its success in operations on all parts of the body all over the world. His predilection was for a saturated solution of boracic acid, and he believed that irritation was caused by the mercuric solution.

Dr. Galezowski, of Paris, France, thought antisepsis necessary in all eye operations, even in chalazion. He had seen in some cases very dangerous results from neglect of this precaution, suppuration of the eyelids, and destruction of part of the cornea.

Professor E. Smith, of Detroit, Michigan, wished to speak on both sides of the question. He thought much was due to the cleanliness in operations. He dipped his instruments in alcohol, used saturated solution of boracic acid, and 1 to 5,000 bichloride. He believed that he had a short time ago saved an eye by antisepsis when it had been exposed several hours the day after operation in the immediate neighborhood of a suppurating surface.

Dr. Power, in closing the discussion, said that the remarks of some of the speakers had been somewhat away from the subject of the paper as he had intended it. He drew attention to the propagation of pure cultures from kerato-iritis, and the production of the disease in rabbits.

The practice of dipping instruments, etc., in solution of boracic acid was followed by him in his hospital practice invariably. He was inclined to think that sublimate in the proportion mentioned is painful. Dr. Baldwin's washing he considered to be really antisepsis. He thought Dr. Galezowski's case of suppuration after chalazion operation was exceptional. He very rarely uses a cataract-knife twice, but prefers to get a new and sharp one from the maker for each extraction. Sharpness and cleanness of cut are essential to a good result.

Professor P. D. Keyser, of Philadelphia, Pennsylvania, read a paper on

OPERATIVE TREATMENT OF SYNECHIÆ POSTERIOR.

He stated that synechiæ were dangerous to the eye, as a rule, in proportion to the firmness of the adhesion. The danger consisted in the recurrent attacks of iritis caused by the dragging and stretching of the part, resulting in new adhesions, cyclitis, and perhaps atrophy, or sympathetic trouble in the other eye. Of operative procedures, he considered removal of part of the iris not advisable where the center of the capsule is transparent. After reviewing the various methods in use for breaking up the offending attachments, of which he believed Streatfield's method to be the most successful with a careful and skillful operator, he spoke of their dangers. He objects to Weber's hook, as being too large to manipulate easily and requiring a large opening in the cornea as well as the other methods with loss of aqueous, which prevents contraction of the iris. He exhibited a delicate modified hook, which he had used with success for a number of years. The method employed is that recommended by Streatfield, and a small opening is made with any cutting needle, affording opportunity of using the hook with as little loss of aqueous as possible. The adhesion having been broken up, atropia is instilled, and a simple Liebriech bandage applied.

Dr. Galezoswki remarked the operation was a very beautiful one, and in many cases afforded good results. He had in five cases of anterior synechia done an operation similar to this, but with a larger hook. The trouble that occurs afterward is that fresh adhesions are formed, and as well after iridectomy, which may go on to the production of cataract or irido-choroiditis. He had seen in many cases, after iridectomy, lymph exuded and vision destroyed. If the inflammatory action continues, he advised acupuncture and antiphlogistic measures, and when it has subsided then do the iridectomy, after two or three weeks.

---

### SECTION ON PUBLIC AND INTERNATIONAL HYGIENE.

SECOND DAY—TUESDAY, SEPTEMBER 6TH.

Dr. J. A. S. Grant (Bey), of Cairo, read a paper with the title,

THE HISTORY OF HYGIENE IN MODERN EGYPT, in which he discussed the methods of hygiene as practiced by the Egyptians under the Viceroy Mohammed Ali and under the Khedive Tewfik Pasha, and as influenced by English dominion; to this he added some critical remarks and practical suggestions.

Dr. Grant also read a paper,

DENGUE IN SYRIA, written by Dr. John Montabet, in which, and also from the discussion, it appeared that there was no essential difference as it occurs in that country and in the United States.

THE RELATION OF STATE MEDICINE TO MEDICAL JURISPRUDENCE

was the title of a paper read by Dr. W. L. Schenck, of Boise City, Kansas.

Dr. Richard H. Day, of Baton Rouge, Louisiana, read a paper in which he made

A REPORT OF AN INQUIRY INTO THE FACTS RELATING TO THE EFFECTS OF OVERFLOW OF THE MISSISSIPPI RIVER.

The paper contains facts and deductions obtained by addressing letters of inquiry to five hundred physicians residing in southern localities. It shows (1) that overflows, as a general rule, are injurious to the public health; (2) that they are more or less injurious according as the inundations are late or early in the season, and whether of long or short duration; (3) that their evil effects upon health are lessened or entirely antagonized by good natural or artificial drainage, and by copious showers of rain occurring during the period of subsidence of the waters; (4) that rice culture is inimical to health only by reason of the improper and insanitary manner of its cultivation; (5) that, as a rule, it is perhaps true that the colored race is less susceptible to the injurious effects of overflows, and of marshy and malarial soils, than the white race. The writer then advises:

(1.) The use of rain-water, stored in large cisterns, both for drinking and cooking purposes.

(2.) That all swamp and wet soils subject to overflows, before being cultivated or settled upon, be cleared of trees, underbush, etc., to admit freely the sun's rays and the free circulation of the winds, and that thorough drainage be effected, so that rapid drainage and drying of the soil be not impeded.

(3.) That in the culture of rice, the common plan of keeping the fields covered for long intervals with stagnant water should be avoided, and that, in lieu thereof, frequent irrigation with fresher and purer water should be adopted, the growing crop being supplied thus as often as needed, and ditches so constructed as to let off the superincumbent water rapidly.

(4) That to promote the health of laborers and residents in the river-deltas and lowlands the dwellings should not be less than four feet from the ground, the floors laid tight, and the doors and windows arranged to afford free ventilation, with galleries on all sides wide enough to prevent beating rains from wetting the rooms; that the houses should be erected on elevated ridges, so that water will not settle under or around them; that only a few shade-trees be allowed to grow to break the force of the direct rays of the sun, and all brush and undergrowth be removed, so as to facilitate the free movement of currents of atmosphere.

Finally, the writer advises the importance of rigid cleanliness of person and surroundings, and strict observance of general sanitary rules. The paper further showed that under improved methods of rice culture and management of lands subject to overflows, the frequency and virulence of disease incident to these localities had been notably diminished.

### SECTION ON DERMATOLOGY AND SYPHILOGRAPHY.

### Second Day—Morning Session.

#### STUDIES IN HIRSUTES.

Professor G. H. Rohe, of Baltimore, Maryland, read a paper on this, in which he directed attention to the theory of natural selection to account for an occurrence of an abnormal development of hair—a reversion of the system to the natural ancestral type. He referred to the work in this field of Dr. Unna, who had shown that the antenatal type of hair, that which falls out soon after birth, and is replaced by a stronger, firmer growth, and accounted for the excessive development by the non-occurrence of this natural order.

Excessive hairiness in one race may not be considered hypertricosis, when it would be in another. The principal interest for the dermatologist centers in the excessive development of hair upon the face, for which we have a sovereign remedy in electrolysis. The reader went over the histories of the noted hairy persons, giving many interesting facts regarding them—a remarkable feature in these cases is an abnormality in the development of the teeth. Usually only few teeth exist, and Darwin has noted the same feature in abnormally hairy dogs. The suggestion was made that the hairy growth in excess might be a compensation for the lack of dental development. Unna's theory of an arrest of development, though attractive, the author does not regard as proven.

Heredity, usually from the male line, is found in almost all cases in universal hirsuties, and many cases of hypertricosis can be explained in the same way.

He related instances in his own practice where the children of hairy-faced parents also showed beards.

That the appearance of the growth is first noticed about the time of puberty is not singular, for other hereditary traits are developed at this time as well.

Dr. Unna, of Hamburg, Germany, regarded the paper as prudent, so far as the pathological portion was concerned. He stated that he upheld his theory, which he had put forth as a more scientific one than other theories which had up to that time been advanced.

He had not been able to study the skin histologically. This is the only way in which advances can be made. And he urged those who have an opportunity to make such studies.

The direction of hair-development changes after birth, and it would be interesting to secure portions of skin to examine for these changes in the different layers of the skin.

It is certainly an hereditary disease, but scientifically this is not a sufficient explanation.

Dr. Thin, of London, England, said he had looked upon the disease as due to a deficient physiological development.

There seems to exist a necessary proportion between the length and breadth of the body and the length and breadth of the hair, and any deviation from this proportion constituted an abnormality. The perfect man develops hair in some parts and little in other parts. That hairy persons are strong is a fallacy.

Excessive hair upon the limbs indicates weakness and debility.

An imperfectly developed sexual being will produce an imperfect being.

A hairy lip indicates a woman who will not well carry out the process of childbirth, and he looked upon it as a flag of warning, and thought the removal of the hairs might not be such a great boon to humanity on that account. In bearded females there is a want of sexual differentiation.

Dr. Gottheil asked how the reader would explain the development of hair late in life, relating a case of excessive development of hair at the age of twenty-eight coincident with cessation of menstruation.

Dr. Ravogli, of Cincinnati, Ohio, said that the human race could be divided into classes by the nature of their hair. He had noticed that men have a greater development of hair upon the face and about the ears and nostrils at about the age of forty-five than in earlier life, and he believed that up to this time, or until the skeleton was perfectly formed, the calcareous salts were taken up by the bones, and after this period were appropriated by the hairy growth. If hair be burned we find salts of lime in the ash, and he thinks there is a relation between the quantity of calcareous salts and abnormal growth of hair.

Dr. Reynolds said that the question was an intricate one. He thought it an established fact that there are two forms of hirsuties, one hereditary and one produced by other causes. The deficiency of the teeth was not present in all cases. He had examined one of the hairy men on exhibition in this country and had found the teeth fully formed. He had observed sexual derangement in bearded females, and regarded it as bearing such a causative relation to the deformity as the same condition bears to acne.

Professor Rohé, in closing, said there were some cases not explicable on the theory of heredity, and he could not explain such cases as that mentioned by Dr. Gottheil. The statement made by Dr. Thin that excessive hair upon the body should be regarded as a sign of weakness, was new to him, and he was inclined to believe in the popular idea, that the man with plenty of hair upon the body was usually a strong man.

### A NEW METHOD OF TREATING DISEASES OF THE SKIN LOCALLY.

Dr. Valentine Knaggs, of London, England, proposed as a substitute for ointments the employment of emulsions which upon drying on the skin form protective films. He had employed these dressings for the past two years with gratifying results in eczemas and other non-specific exudations. The inunction of the body with fixed oils has been found of immense service, but they are not adhesive, and exuding fluids readily escape from the surface. There are two methods of rendering oily substances adhesive: 1. By adding to them resinous, gummy, or alkaline substances. 2. In making use of gum to combine or emulsify a fat with water.

Adhesive preparations, unlike oils, tend to arrest skin action. Thus tar, varnish, or collodion form an impervious covering. Ointments to which adhesive substances are added diminish skin action, but do not abolish it.

Well-made emulsions resemble milk or cream, are soluble in watery fluids, and are markedly adhesive, and are made by combining an ointment basis with water, a vegetable gum, and a suitable antiseptic.

Thus the formula preferred would read :

℞. Paraffine molle.................. ℥j.

Pulv. gum acac.................. gr. 160

Boracic acid.................. gr. 16

Aquæ.................. ad ℥ij.

Stir until emulsified.

Bismuth, zinc, sulphur, or other medicament may be added as desired. Smeared over the skin, a film is formed, which is flexible and protective, and the antiseptics and medicaments have the best opportunity of exercising a beneficial influence.

The author called especial attention to the fact that all lint and textile dressings could be dispensed with. He did not expect these gum emulsions to displace older methods, but that they would serve a useful purpose.

In discussing the paper, Dr. Unna said that we were now in the period of transition, making our annual change of treatment. If was very well for us as specialists to discuss new treatments, but we should not give them to the general practitioner until we were quite sure that they were good, and especially should not confuse him with new treatments until we were quite sure that they were new. He had worked much with fixed dressings and varnishes, and some years ago had described such a varnish as had the reader of the paper. He was glad to confirm the reader's claim regarding the utility of the emulsion dressing, but could not concede to him that it was new, as there many other varnishes made with gums and fats. He did not believe that any varnish which is soluble in water can prevent a great of evaporation from the skin.

### A NEW METHOD OF TREATING THE VEGETABLE PARASITIC DISEASES OF THE SKIN, by Professor H. J. Reynolds, of Chicago, Illinois.

First, the parasiticide must be applied so as to reach the bottom of the hair-follicles in favus, ringworm of the scalp, and barber's itch. The reader proposed to apply the medicament to the diseased part and place over it the positive pole of an electric battery, and the negative upon some other part of the body, and induce the penetration of the solution to the deeper parts by a well-known law of elected physics. He uses a battery composed of a large number of small cells, such as is used for the removal of hairs. The strength of the current must vary with the sensitiveness of the parts—five to fifteen cells, according to the feelings of the patient. Where there is much hyperæmia the current must be weakened. Cocaine, applied in this way, produces anæsthesia of the whole scalp. The surface is first to be thoroughly cleansed. Then the sponge of the positive electrode is to be saturated with the parasiticide solution and placed over the diseased patch, and the moist negative sponge over the skin of some other part of the body. A one per cent. solution of the bichloride was the parasiticide employed in the cases reported. He had only had an opportunity to thus treat three cases, but all have been so successful as to give him great confidence in its usefulness.

Dr. Thin said he had been impressed with the great variety of remedies found in literature reputed to cure tinea tonsurans quickly. He regarded the strong solution of bichloride of mercury as dangerous to life, and also likely to cause permanent baldness, although it was the remedy most likely to cure the disease.

Dr. Juler, of Cincinnati, Ohio, said in cases of favus we usually expect to find a change in the condition of the patient's general health. He regarded it as an extremely obstinate affection, and was inclined to look upon hasty cures as errors in diagnosis.

Dr. Davis, of London, England, thought that in America skin diseases were more easily cured than in England, from the reports of rapid cures, especially of ringworm and the like.

Professor Rohé thought perhaps the differences in results were due to the differences in application. He thought the bichloride, in solution of 1 to 1,000, sufficient if applied often enough, before the new spores had an opportunity to be produced. Fully developed organisms resist much less than the spores. So long as one spore remains undestroyed, so long will the danger last. The hyposulphite of soda will destroy, in a short time, the organism of tinea versicolor.

Dr. MacGuire, of Louisville, Kentucky, said chrysarobin in gutta percha had succeed well in sixteen cases of tinea trycophyton recently treated by him.

Dr. Ravogli said if epilation was practiced, the parasiticide would reach the spores and the case could be sooner cured.

Dr. Yeamans said the whole matter turned upon whether we can or cannot reach the spores. He believed that it was unnecessary for any case of favus to exist under proper scientific treatment for over four weeks, epilation being practiced daily.

In closing, Professor Reynolds said it was an established fact that cocaine could be applied to the scalp so as to thoroughly anæsthetize the entire thickness of the scalp. Now the question is, whether a parasiticide applied in this way will not act equally well. He thought the electricity might have some influence in destroying the spores.

### SECTION ON GENERAL MEDICINE.

### Second Day—Afternoon Session.

Dr. Josef Körösi, Director of the Communal Statistics of Budapesth, Hungary, read a paper on

THE PREVENTIVE POWER OF VACCINATION, with a critical review of the statistics of vaccination. After gathering all the different modes of argumentation used up to the present time to attack or to defend the protective power of vaccination, he arrived at the result that the statistical base of these proofs is much weaker than is generally supposed by men of science who could not enter into an examination of the value of the different statistical methods. Even when the direct proof could be furnished, and even when we would find in this way that the morbidity or mortality of the non-vaccinated is the greater one, the anti-vaccinators reply that the whole of the non-vaccinated represents a weaker totality, consisting of the poorer elements, the sick and weaker children; and that this totality is naturally more exposed to get any sickness, but not in consequence of the lack of vaccination, but of the lack of vital powers—an argument which threatens to subvert all statistics of vaccination.

Dr. Körösi shows us that this difficulty can be settled. If we knew what is, for instance, the general lethality (Dr. Körösi distinguishes between mortality and lethality. Mortality is the chance for each living person to die; lethality is the chance for those who are already sick to die. The two notions are widely different but, notwithstanding, often confounded. The mortality of small-pox, for instance, rose to one per centum, the lethality might rise to sixty or seventy per centum) of the non-vaccinated, we could learn whether their special small-pox lethality is greater; this difference ought to be attributed exclusively to the lack of vaccination. But to ascertain the general lethality of the vaccinated and non-vaccinated people, it would be necessary that the hospitals should state, not only for the small-pox patients, but also for each case of sickness—even the surgical cases not excluded—whether they were vaccinated or not. This innovation was introduced in 1886, at the proposition of Dr. Körösi, in nineteen hospitals of Budapesth and provincial cities in Hungary. Here the results of the first year's observation, embracing more than twenty thousand cases, showed the general lethality of the vaccinated patients to be eight per centum, and that of the non-vaccinated thirteen per centum.

Thus the assertion of the anti-vaccinators, that among the non-vaccinated the lethality is probably a greater one, is found to be true. But among the small-pox patients the lethality of the non-vaccinated rose to 6.66 per centum; the lethality of the non-vaccinated should thus represent about ten per centum, but in fact that lethality is not less than 49.68 per centum. No doubt it was wrong to regard the whole of this increase (nearly eight hundred per centum) as the consequence of the non-vaccination. The result is a product of two factors; on one hand of the weaker constitution of the non-vaccinated, and on the other hand of the lack of vaccination. Knowing now the value of one factor, we can calculate what has to be attributed to the other. So we can state that for the non-vaccinated small-pox patients the chance of dying is raised *exclusively by the lack of vaccination* to five hundred per centum.

The same observation made it possible to state also in what degree the morbidity of the non-vaccinated is raised. The result is that the lack of vaccination causes three and a half times more cases of small-pox.

To apply the new method on the mortality, it would be required that at each case of death—and not, as is usual, only at the deaths caused by small-pox—it should be stated whether the person was vaccinated or not. This new regulation has been introduced at Budapesth since 1886; the coroners have to inquire the fact regarding vaccination in each case of death. As also nine other Hungarian cities agree to introduce this new regulation, the observations of Dr. Körösi embrace a population of 717,195 persons. The results of the first year show that the chance to die by small-pox is raised by six hundred per centum for each non-vaccinated person exclusively in consequence of the lack of vaccination. This new and fruitful method of investigation has finally been applied also to the very important problems of inoculation of syphilis, erysipelas, tuberculosis, etc. In the ordinary statistics of vaccination it is nearly impossible to answer this question. If a man who has been vaccinated in his childhood gets tuberculosis later, how could we know whether his sickness had been caused by vaccination or any other cause? But the new method furnishes the answer. If tuberculosis, syphilis, etc., have been caused by inoculation we ought to find among the vaccinated people more cases than among the non-vaccinated. It is now ascertained that—at least in the first year of observation—there was to be found no influence of vaccination upon syphilis or tuberculosis. But what he found was that for the children the number of the cutaneous diseases is raised by vaccination, but only by thirteen per centum. Making up now the balance of vaccination and counting its benefits on the credit, its damages on the debit side, it is found that among a population like that of the United States the vaccination would save annually 120,000 lives, while the number of children dying from cutaneous diseases, etc., caused by vaccination, might make 300, so that the balance is an extremely favorable one. Vaccinating is an operation, but who would prohibit a life-saving operation where the chances of danger are so extremely few as above mentioned?

Dr. Körösi finished his paper with the remark that after having now furnished direct answers on the protective power of vaccination, we might safely extinguish doubts about the blessings of Jenner's important discovery, which, after the ingenious generalizations of Pasteur, has lost the character of an inconceivable and quite extraordinary appearance, and which must be regarded as one of the most beneficial results of happy empiricism and science.

Dr. C. A. Leale, of New York, N. Y., expressed himself as greatly interested in the paper, especially for the valuable statistics, evidently the result of so much patient labor. He referred to a commission which had been appointed under his supervision to inquire after the health of the sick children in New York, with the result that there was not a case reported which had had small-pox. This he attributes to the good virus and enforcement of vaccination among all classes.

Twenty years ago he was called to investigate two cases of death alleged to be the result of small-pox, (?) but these were evidently from erysipelas due to a scratch; this was rendered more positive, as others were vaccinated with the same virus without harm. He once saw a child where it was certified that the child was suffering from the secondary stages of syphilis due to vaccination.

Dr. W. M. Whitmarsh, of England, inquired what virus was used, humanized or bovine.

Dr. Körösi said it depended upon the physician.

Professor John F. Lynch, of Baltimore, Maryland, said that it was only cranks who will contend that vaccination does not prevent and modify small-pox. Animal virus will always be safe and ought always to be used.

A vote of thanks was extended to Dr. Körösi, and a committee appointed to consider his paper.

Dr. Körösi said that statistics reported against vaccination were greatly falsified.

Dr. W. M. Whitmarsh, of London, England, read papers on

VACCINATION AND ON PASTEUR'S TREATMENT.

He first considered the subject of vaccination, giving a *résumé* of its history from the time of Jenner. He was not in a position, even from a very large experience, to state that vaccination is a preventive against variola, although many believe it to be so. Vaccination does, however, lessen the liability, even though it may not give a complete immunity from disease. Forty years ago sixty per centum of the people in England were to be seen pitted by small-pox, while at the present day there is hardly one per centum.

In England it is compulsory that all children over three months old shall be vaccinated. Such a law being in vogue, it is the duty of the state to use special care in the selecting of good lymph. This evidently has not been done, the authorities at present depending upon humanized virus, which they dealt out gratuitously to the poor.

That vaccination may transmit disease is conclusively proven in the case of Dr. Cary, who syphilized himself in endeavoring to prove that such a transmission was impossible. Again, there were the instances of the large number of children in Sweden, and the body of soldiers in Algiers, who were similarly infected.

Educate people up to the fact that vaccination, when properly performed, is a good measure, and then their common sense will make them adopt it. During the year ending September, 1885, there were in England 2,806 persons prosecuted for refusing to comply with the law and be vaccinated.

As to how many punctures should be made, and the frequency with which the vaccination should be repeated, there is a variety of opinions. It seems probable that seven years is the longest time allowable before revaccinating.

PASTEUR'S METHOD.

In the paper entitled "Pasteur's Treatment" he reviewed very elaborately the method and principle involved in the preparation of the virus for inoculation. The various instruments used were presented, and their use explained; also a solution, hermetically sealed, containing the prepared virus. Sterilized beef-tea is the vehicle used to contain the virus.

As to Pasteur, he is undoubtedly a first-class scientific chemist, but doubtless he knows little of the art of surgery or the science of medicine. So far as his operations are directed he is constantly changing his methods, thereby showing that he is somewhat doubtful of his standpoints.

It is proved without doubt that dogs shut up in a room and given but a small quantity of water will go mad. In the cases presented and operated upon in Pasteur's laboratory it is extremely doubtful as to whether the patients have had hydrophobia, and consequently it is difficult to determine the curative efficacy of his inoculations. Pasteur originated a disease in rabbits, which he called rabies. It is important, since some of his cases have been fatal, that the disease be not originated, by this process of treatment, in a person supposed to be, though not actually, suffering from the disease.

There were cases in which Pasteur's treatment had failed, even in a case in which there had been no delay in coming under the treatment.

The only way to settle the question is by the means employed fifty years ago in reference to small-pox, and that was upon convicts, allowing them their choice between their sentence and becoming the subject of experimentation.

It is also necessary to enforce more stringent laws in reference to dogs. They ought to be registered, muzzled, and more carefully looked after by examining veterinary surgeons.

Great credit is due to Pasteur for his earnest labors, and it is to be hoped that, if he has not already, he may eventually make a new discovery.

Dr. C. A. Leale, New York, N. Y., expressed his great interest in the papers, and spoke on the latter. As medical officer for several large benevolent institutions for sick children he had coming under his observation from 18,000 to 20,000 sick children annually. These children were sent from all parts of the city, and all were carefully examined by competent medical men. As these children are only the sick ones of the families, they represent the number taken from at least 100,000 children in the city and surrounding places. He had never seen, or had reported to him, a single case of hydrophobia, although hundreds of these children had at different times been bitten by dogs. He had had similar experience in dispensary practice.

He is convinced that much harm has been done by unnecessarily exciting those bitten. It is to be sincerely hoped that the profession will not, with our present knowledge, resort to Pasteur's method of inoculation.

Dr. W. Welch, Philadelphia, Pennsylvania: Believes vaccination possesses the power of absolutely preventing small-pox. To be entirely free from danger it must be recently and properly done, and under such circumstances he had never seen any bad result. In his hospital experience he had had 5,000 cases come under his observation, and after vaccination he allowed attendants to wait upon the sick patients without their contracting the disease. Humanized virus is preferable, especially such as has been long humanized through several inoculations. It is well, with long humanized virus, to make several inoculations, as virus thus attenuated does not make so profound an impression.

As to durability, it does run out. It was noticeable that among the cases admitted there were no, or very few, children brought in with the disease who had been vaccinated. One of these died.

The dangers of vaccination are two: syphilis and erysipelas—the latter often being caused by carelessness on the part of the physician.

---

### SECTION ON OBSTETRICS.

### Second Day—Afternoon Session.

Dr. James C. Cameron, of Montreal, Canada, read a paper entitled

THE INFLUENCE OF LEUKÆMIA ON PREGNANCY.

In this he showed, by a *résumé* of the literature, how incomplete our knowledge of the subject still is. We knew that cases were most frequent in women, especially during pregnancy or at the climacteric. Its effect upon the reproductive organs was but little known, and barely mentioned in any work. The disease was apt to begin during the latter part of pregnancy, and indeed, in many of those who became sallow and anæmic during pregnancy, though only temporarily so, the ratio of white to red blood-corpuscles was much increased.

A case which he reported was unique, in that pregnancy recurred successively during the progress of the disease, and was also interesting in showing a marked hereditary tendency—the parents of the patient and her six children being all leukæmic. The splenic tumor was first noticed during a pregnancy, and increased markedly in size during each successive gestation, the disease running a remarkably chronic course. A fact worthy of note, was that the red blood-corpuscles of a child born when the disease was well marked, were in the normal proportion in the vessels of the child, even above the normal in the placental artery, but much diminished in the placental vein, showing that the blood actually lost red corpuscles while passing through the placenta. In the placental sinuses the red globules were fewer than in the general circulation of the mother.

Professor Charles Warrington Earle, of Chicago, Illinois, recalled two cases of extreme anæmia, together with great emaciation, occurring during pregnancy, and which at the autopsies were found to be caused by chronic inflammation of the pancreas, all other organs being normal. The pancreas was white and hard, and the microscope showed a great increase in the connective tissue. He thought that this condition might explain some cases of pernicious anæmia, and proposed for it the name of pancreatic anæmia.

Professor A. Charpentier, of Paris, France, read a paper entitled

L'UREMIE EXPERIMENTALE.

In this he detailed the results of certain experiments made by him on the artificial production of uræmia in gravid animals, by the injecting into the blood, at intervals, of urea until it was present in excess. In these cases the death of the fœtus preceded that of the mother, and the quanty of urea in its veins was in excess of that in the mother's. The death of the fœtus was caused by this excess of urea in its circulation.

Professor W. T. Lusk, of New York, N. Y., thought the paper illustrated a most important point. The explanation ot the manner of death of the fœtus was certainly ingenious. He had supposed that its death was due to the presence of carbonic dioxide in the blood of the mother.

Dr. Duncan C. MacCallum, of Montreal, Canada, said that in marked albuminuria with severe symptoms, we were justified in inducing premature labor, and if the death of the child was apt to occur, as had been shown, it was another strong reason why we should not delay too long.

Professor Alexander R. Simpson, of Edinburgh, Scotland, read a paper giving the results of his efforts to secure

UNIFORMITY IN OBSTETRICAL NOMENCLATURE, presenting a schedule showing the proposed changes, and detailing some of the opinions he had received from eminent obstetricians. The subject had been opened at the Eighth International Congress, and he hoped this one would take some definite action in the matter.

After animated discussion, a committee, consisting of Professors Miller, Simpson, Lusk, and King, was appointed to consider the matter, and report on Friday, the 9th inst.

Professor William T. Lusk, of New York, N. Y., presented a paper on

THE PROGNOSIS OF THE CÆSARIAN SECTION.

If it were proposed to beat out the brains of a living child, the suggestion would be received with horror, no matter how great the the surgical emergency might be, and yet, with the child unborn, craniotomy was often done for insignificant reasons. A careful *résumé* of the statistics showed very favorable results, even with the old method, when the surroundings were favorable, and the operation was performed with proper surgical skill and accessories. Death was most often the result of an avoidable cause. The reader compared the brilliant results obtained abroad with the mortality attending recent American operations, and thought that a more favorable prognosis could not be expected until we had learned to recognize the conditions requiring the operation before the time when it should be done.

Defective diagnosis was the great bar to progress in this country, and all practitioners should qualify themselves to recognize the various degrees of pelvic deformity. An operator should possess at least a theoretical knowledge of the technique of the procedure, and while it was not desirable that any one should attempt to perform the section, men capable of doing the operation could usually be found, even out of the larger cities.

The paper closed with a plea for Cæsarean section as opposed to craniotomy, even in pelves with a conjugate of three inches.

Dr. M. Sänger, of Leipsic, Germany, presented a paper on

THE CÆSAREAN OPERATION, which was read in abstract.

Sanger's operation was to be preferred to the Porro when the child was living and could not be delivered by any other operation, or when the child was dead and could not be delivered by craniotomy or embryotomy, or could be so delivered only with the greatest danger to the mother. Good results could be obtained by the Cæsarean section only under certain conditions and when the operation was performed according to certain approved technical principles. These conditions are: 1. The maintenance of an aseptic condition in the uterine cavity, and, 2, early performance of the operation.

Sänger thinks that the cause of the greater American mortality is delay, and only trying the section when other operations have been unsuccessful. He lays stress on the following:

(1) Antiseptic precautions as in other laparotomies.

(2) The abdominal incision should be made through the linea alba over the middle of the fundus, about sixteen centimeters long.

(3) It is not advisable to evert the unopened uterus, as it requires a large incision, except where the fœtus is dead or there are not sufficient assistants.

(4) The elastic ligature is not to be used before the uterus is opened, as it endangers the life of the child, or may incarcerate parts of the child, so that it may have to be loosened at a time when the operator requires his hands for more important matters.

(5) The uterus is to opened in situ, by a frontal median incision; cut through placenta, or push it to one side; extract child by the legs; if head is caught, extend incision upward, to prevent any downward laceration of the uterus. At the same time, an assistant is to press the abdominal walls toward the uterus to prevent prolapse of intestines or flow of fluid into the abdominal cavity.

(6) The danger from hæmorrhage is not so great as is commonly supposed. By pressure on the inferior segment, and by slight torsion or flexion of the uterus and broad ligaments, the bleeding can be much lessened. Elastic ligature is to be avoided if possible.

(7) Care must be taken in regard to three points in suturing; 1. Accurate union of the incised surface of the uterus by numerous sutures, where by a broad and close union is obtained. 2. Avoidance of suture-canals in the uterine cavity. 3. Especially careful union of the serous surfaces. Silk is preferred to silver-wire, because silk can be absorbed. Excellent results can be obtained with catgut prepared in oil of juniper, chromic acid, or mercuric bichloride.

Sänger's indications for the Porro operation are: When the flow of secretion from the uterus is impeded by stenosis of the cervix or vagina, or, in some cases of tumors of the corpus uteri, myomata, etc. He prefers Cæsarean section where the myomata are retro-cervical or retro-vaginal, because the removal of the whole mass is then impossible or dangerous and the removal of the uterus does no good. In osteomalacia he prefers the Cæsarean section with removal of the ovaries to the Porro operation. He takes exception to Martin's recommendation to do the Porro operation in cases in which the puerperium becomes dangerous to the patient, as in far-advanced affections of the heart or lungs. He thinks such a case has as good a chance of recovery after Cæsarean section as after Porro's operation.

Professor W. H. Wathen, of Louisville, Kentucky, read a paper on

ABDOMINAL SECTION FOR REMOVAL OF THE FŒTUS.

He urged that abdominal section should be done in all cases, instead of craniotomy, where the child was living. Craniotomy was often unnecessarily done. The maternal mortality was not greater in selected cases of Cæsarean section than after craniotomy. In moderate degrees of pelvic contraction we should induce premature labor, if the condition was discovered early. Abdominal section should become an operation of election and not a last resort. The uterine suture and careful toilet of the peritoneum were indispensable.

---

### SECTION ON PATHOLOGY.

### Second Day.

The president read a paper on

THE PATHOLOGY OF REYNAUD'S DISEASE, and reported an unusual case.

Dr. E. O. Shakespeare, of Philadelphia, Pennsylvania, followed with a paper entitled

EXPERIMENTAL RESEARCHES CONCERNING THE INFECTIOUS NATURE OF TRAUMATIC TETANUS.

Professor Henry Sewell presented a paper on

EXPERIMENTS ON THE PREVENTIVE INOCULATION OF RATTLESNAKE VENUM, which was read by Professor Vaughan, of Ann Arbor, Michigan.

Dr. De Solomon, of Michigan, read a paper on

THE PRODUCTION OF IMMUNITY BY THE HYPODERMIC INJECTION OF STERILIZED CULTURES.

---

### SECTION ON PHYSIOLOGY.

### Second Day.

Dr. W. D. Halliburton, of London, England, read a paper entitled

COMPARISON OF THE COAGULATION OF THE BLOOD WITH RIGOR MORTIS.

The coagulation of the blood can be prevented either by cold or by the admixture of certain neutral salts. Kühne was the first to show that cold prevents the coagulation of the muscle plasma of frogs. By means of methods very similar to those employed by that observer, the muscle plasma of warm-blooded animals can also be prevented from coagulating by the same agency. Mixture of the muscle plasma with solutions of certain neutral salts will also prevent its coagulation; and, as in the case of salted blood plasma, dilution of such salted muscle plasma brings about the formation of a clot of myosin; this occurs most readily at the temperature of the body, and is entirely prevented by a temperature of 0° C. (?) It is hastened by the addition of a ferment prepared from muscle in the same way as Schmidt's fibrin ferment is prepared from blood. Rigor mortis is thus the result of a ferment action similar to that which converts fibrinogen into fibrin; but the myosin ferment is not identical with fibrin ferment, as it does not hasten the coagulation of salted blood plasma, nor does the fibrin ferment hasten the coagulation of muscle plasma.

The myosin ferment is either identical with, or closely allied to, one of the proteids of the muscle plasma, which has the properties of an albuminose.

Though it is thus seen there are very striking resemblances between the formation of fibrin and that of myosin, there are also certain differences, of which the most important are the three following:

1. Myosin can be easily re-dissolved by solutions of neutral salts, and this solution can be made to undergo re-coagulation by dilution and addition of myosin ferment. The passing off of rigor mortis seems to be due to a similar reconversion of myosin into myosinogen. Fibrin, on the other hand, cannot be made to undergo such a re-coagulation.

2. The formation of myosin is accompanied by the formation of lactic acid. The same acid is formed when the re-coagulation of pure myosin occurs, and its source is thus shown to be a proteid one, not a carbohydrate, as some have supposed. Fibrin formation is not accompanied by the development of any acid, so far as we are aware.

3. The conversion of myosinogen into myosin is not accompanied by the formation of another globulin, as is the case in the conversion of fibrinogen into fibrin.

Dr. Thomas W. Poole, of Lindsay, Ontario, read a paper entitled.

THE NECESSITY FOR A MODIFICATION OF CERTAIN PHYSIOLOGICAL DOCTRINES REGARDING THE INTER-RELATIONS OF NERVE AND MUSCLE.

1. He hoped to show, on the highest physiological authority, that muscles of the involuntary class pass into a state of spasm or contraction, not when their motor nerves are stimulated, but when these nerves are cut, or are paralyzed, or dead.

2. That the same is true of the muscular bands of the arterial coats.

3. That the statements given in our physiological works as to the excited condition of the nervous centers in such states as asphyxia, and the theories of Traube and others in explanation of the Cheyene-Stokes' respiration, in which impure, venous blood, loaded with carbonic acid and deficient in oxygen, is assumed to stimulate and excite the nervous centers, are absurd, and have been put forward solely to meet the exigency of an erroneous theory.

4. His thesis entered into what he deemed conclusive evidence that electricity, as employed for physiological and therapeutical purposes, is not a stimulant but a paralyzing agent to nerve-activity, and that the undoubted beneficial effects of this agent may all be satisfactorily accounted for in strict accord with its role as a paralyzer.

5. The same applies also to strychnia.

6. The action of the vascular sedatives, aconite, ergot, etc., and the rigidity and subsequent relaxation of the muscles during anæsthesia are amply accounted for on the view presented in this paper.

7. This theory is not an extension of the inhibitory hypothesis of recent physiology.

8. Ample proof is produced that "irritation" and "inflammation" are not attended by excitation of nervous activity, but the reverse; also that what is called "morbid" nerve-force, as something different from proper nerve-function, does not exist.

9. Even the voluntary muscles pass into a state of spasm or contraction under conditions which may properly be regarded as attended by a deprivation of nerve-force, oftener, perhaps, than is generally supposed, as in cases depending on "irritation," which is a paretic condition rather than one of exalted nervous activity.

10. It is not necessary to his thesis that he hold or prove rigor mortis to be a condition of muscular contraction. But this state has not yet been otherwise satisfactorily explained, the myosin hypothesis proving unsatisfactory in that respect.

---

### SECTION ON OPHTHALMOLOGY.

### Sedond Day—Afternoon Session.

The first paper read was by Dr. A. Mooren, of Düsseldorf, Germany, on

#### THE SIMPLEST METHOD OF CATARACT-EXTRACTION.

He had had an experience of 5,019 extractions since the beginning of his ophthalmological practice in 1855. He had not invented any new instrument. With the progress of antisepsis and anæsthesia, there was less need than formerly of dexterity of manipulation. He regarded cleanliness and disinfection as clearly identical. He avoided the use of carbolic acid on account of its irritant effect. Preparatory to the operation, he washes the whole face of the patient, then instils a few drops of cocaine. Immediately before operating, he washes the everted eyelids with a three per centum solution of boracic acid. The operation may be done without a speculum. The only instruments necessary are a Graefe knife, and simple forceps to fix the eyeball. The cut is made downward, and the same knife is used to lacerate the capsule through the intact pupil. Fixation should be abandoned when the patient is restless. Deliver the lens by gentle stroking, then remove the speculum, and rub the closed eyelids over the cornea, and wash again with the same solution of boracic acid. Then a light compress is put on, and retained by means of a strip of adhesive plaster, passing from temple to temple. The plaster should not be allowed to remain longer than four days, on account of its irritant effect upon the skin. In high degrees of atheroma it is better to do an iridectomy, as there may be a resulting leucoma. In such cases Dr. Mooren does the combined operation, making the iridectomy upward. In another form of corneal extraction he uses a lance-shaped knife to make the incision and open the capsule. Here he fixes the eye at the lower margin of the cornea. The iris seldom protrudes, and if it does, cut it off. In this form of extraction, if traumatic cases be eliminated, there is scarcely any bad result. He spoke of modifications in procedure, where the cataract has a hard, large nucleus, and of the necessity for an iridectomy where a foreign body was in the lens, to guard against its being brushed off by the iris during extraction and left in the eye.

Förster's method of ripening cataracts for a few years back has been practiced by Dr. Mooren. It consists in puncture of the anterior chamber and sufficient rubbing of the cornea through the closed eyelid. The introduction of instruments into the eye is not necessary. These cases, when complicated with choroiditis, require to be treated with great caution. One case required a year to mature, and a second had an extraction of a half clear lens fifteen months after the puncture, while a third failed entirely to ripen. The after-treatment is complete physical and intellectual rest.

In cases complicated with constitutional diseases, of course, general appropriate treatment is necessary.

Dr. Galezowski, of Paris, France, prefers extraction through a dilated pupil without iridectomy by corneal section. He makes a sclero-corneal puncture and counter-puncture, bringing out the knife so as to form an ellipsoid incision. With the same knife he makes a vertical incision in the capsule from top to bottom. Pressure below without the blepharostat extrudes the lens.

The incision is made in the upper half of the cornea. The accidents are (1) hernia of iris after operation; (2) suppuration of the cornea; (3) iritis; (4) secondary cararact. To prevent hernia iridis do not bring the incision too far out toward the sclero-corneal margin. Then do not open the eye too frequently. If opened every day after operation it makes the patient contract the lids and press out the iris. Do not open the eye for perhaps five or six days; only remove dressing to note swelling and condition.

To meet the second indication a disk of antiseptic gelatine is introduced into the eye under the eyelids, the lids closed over it, and left so. These disks are permeated with sublimate and cocaine. In three hundred cases, only two have had suppuration.

For the astigmatism existing after cataract-extraction he accounts in this way: In almost all eyes astigmatism exists both in cornea and lens, that of the lens correcting or neutralizing the corneal aberration. When the lens is removed this correcting element is lost, so that astigmatism is found where it did not appear before operation. If the capsule is hard or tough it is seized with a pair of forceps, drawn within reach and cut off with scissors.

Professor N. Monolescu, of Bucharest, read a paper on

CATARACT-EXTRACTION WITHOUT, AS COMPARED WITH EXTRACTION WITH, IRIDECTOMY.

In his simple operations he had trouble from prolapse of iris and other complications due to the difficulty of cleaning out the chamber, and he believes the combined operation to be the more reliable, as it lowers the danger from retained matters and iritic complications. He uses corrosive sublimate as a wash, the toothed forceps for removing the anterior capsule, and closes the wound with a rubber spatula. The eye is dressed with a firm close bandage, and he changes it early.

Dr. E. Landolt, of Paris, France, remarked that there never will come a time when only one operation will be the best for all cases of cataract. He himself was greatly in favor of the combined operation, and thought it was better rather to seek a fair degree of useful vision than a symmetrical pupil.

Dr. Abadtie, of Paris, France, finds that extraction withou iridectomy may sometimes complicate the reparative process, and it may be difficult to remove cortical substance. He reserves the operation for cases that are healthy, fully ripe, and which promise ease of extraction and good recovery.

Dr. Marmion, of Washington, D.C., said that his faith in iridectomy in cataract-extraction had not been shaken by the arguments that had been advanced.

Mr. H. Power, of London, England, acknowledged himself a convert to the operation without iridectomy. Three years ago he had thought he should never change his opinion or method. But cocaine had rendered accidents less likely and given the patients more confidence. He made his incision nearer the sclero-corneal junction than Dr. Galezowski, but agreed with him that the less the eye is looked at the better. A very light compress is to be applied over the eye after operation. If suppuration occur, attend to the general health. In regard to iritis, he did not think that iridectomy was any protection against it. In regard to secondary cataract, he had it occur just as many times without as with iridectomy. If prolapse of iris occurs during the operation, cut it off.

Professor D. S. Reynolds, of Louisville, Kentucky, thought that in cocaine and without general anæsthesia we had eliminated the chief sources of failure in cataract-extractions. In carefully selected cases simple extraction might be preferable. In such cases he uses Graefe's knife, and allows the aqueous humor to escape before completing the section, which he makes as nearly as possible in the plane of the anterior face of the iris. In the combined operation he uses Beer's knife. He attaches importance to the way in which the capsule is divided. He makes a flap opening upward, and it frequently tears in other directions. As to eserine after extractions, if the pupil remains large, use it; if small, do not. He never uses fixation-forceps, but steadies the eye with his fingers. He has no use for an assistant in a cataract-extraction. The dressing is a minute film of absorbent cotton, held in place by a strip of adhesive plaster from lower lid to brow, about an inch wide and one inch and a half long, and renewed daily.

Professor P. D. Keyser, of Philadelphia, Pennsylvania, objected to one operation for all classes of cases. Before the introduction of cocaine he made the downward section; now he makes the cut upward. He tries to remove a piece of the capsule. He never operates without examining the urine for albumen. If there is albumen he looks out for iritis. He uses a solution of boracic acid, as sublimate seems to irritate the eye.

Dr. J. L. Thompson, of Indianapolis, Indiana, found that malaria complicates and might cause iritis after extractions.

Dr. Baker, of Cleveland, Ohio, concluded that he could not do without an iridectomy. He follows Mr. Wolf's (of Scotland) suggestion, and makes a small iridectomy, leaving a small pupil, delivers the lens slowly and avoids touching the cornea, saving the patient pain after the operation.

Dr. E. Landolt prefers to make a large iridectomy if he has a large corneal section, and excise as much iris as he can. A small iridectomy is more dangerous than a large one, which allows one to control the field of operation better. He had not had any irritating effect from sublimate solutions in his cases.

Professor S. M. Burnett, of Washington, D. C., has extracted twenty to twenty-five cases without iridectomy, one of black cataract. He thinks there is no form of cataract that cannot be operated on by this method, and there are no more complications than where iridectomy is made. The removal of cortical substance is more difficult without than with iridectomy. He washes out the cortex.

Professor E. Smith, of Detroit, Michigan, thought there was no certain operation that could be laid down for all cases. He found that it was not always convenient to do a preliminary iridectomy some weeks ahead, as the patients objected to more than one operation.

Dr. Beaver, of Reading, Pennsylvania, asked whether the simple extraction was applicable in weak and flabby corneæ that fell down in wrinkles when the section was made.

Dr. Valk, of New York, N. Y., has had made an instrument which combines both methods to some extent. He makes a section, then introduces his retracting forceps, which he exhibited, and tucks the iris back out of the way, delivering the lens over the arms of the forceps. The iris is then allowed to fall back to its place, no violence being done it, and giving a perfectly round pupil with no iritis.

The president, Professor Chisholm, wished that more had been said on the subject of the introduction of antiseptic fluids and eserine into the eye. He submerges the eye in sublimate solution, or one of biniodide of mercury, makes an iridectomy not as large as the corneal section, and simply closes the eyelids and fastens them together with a strip of diaphanous adhesive plaster clear enough to count the eyelashes through, and if no discharge appears leaves it undisturbed.

Dr. Galezowski, in closing, said that he recognized the two classes of cases, in one of which the simple operation was all that was necessary, while in the other the combined operation was the better adapted.

Mr. H. Power said in regard to antisepsis that he always uses a knife, and a sharp one, rarely using the same knife for an extraction more than once. He believes the introduction of fluids into the anterior chamber might be dangerous, and he has not seen benefit from the instillation of eserine.

---

### SECTION ON OTOLOGY.

### Second Day—Afternoon Session.

A paper was read by Professor T. E. Murrell, of Little Rock, Arkansas, on the

### PECULIARITIES IN STRUCTURE AND DISEASES OF THE EAR OF THE NEGRO.

By negro was meant the typical African, and not the mixed races. The negro has fewer aural affections than the white man, and for this reason deafness is much less common in this race. His immunity from aural diseases is due to other than accidental causes alone. Peculiarities in anatomy are met with sufficiently striking to explain some of his exemptions. The following peculiarities in the anatomy of the ear and adnexa are more or less conspicuous: The pinna is small, with lack of development above the concha, and is set closely to the side of the head. The external auditory canal is large, quite straight, and of less average depth than the standard. The membrana tampani is large in its diameters, and forms a less oblique angle with the axis of the canal than that usually given. The mastoid process is very slightly developed, and inconspicuous. The pharynx is large and capacious, with great breadth between the fauces. The nares are broad and flared, giving immense breadth to the choanæ. Deflection of the septum is seldom an obstruction to nasal respiration. Diseases of the external auditory canal are mostly accidental and traumatic. Impacted cerumen is less common than in the white races. Otitis parasitica is rare. Otitis media suppurativa acuta is rather frequent in children, but less so than in whites. Rhinitis purulenta is common in strumous children, and in these the suppuration in the middle ear mostly occurs. Recovery generally takes place with little loss to hearing. Otitis media suppurativa chronica is still less common. Mastoiditis is extremely rare. Otitis media catarrhalis chronica is very infrequent.

Deaf-mutism is of much smaller percentage than in the white races. Among the accidental causes of aural affections may be mentioned: The southern negro lives in open houses, and is therefore less liable to head colds than otherwise. His colds are mostly pectoral.

#### DISCUSSION.

The paper gave rise to discussion, and as the subject was a new one, to diversity of opinion.

Dr. C. M. Hobby, Iowa City, Iowa, thinks the excellent paper of Professor Murrell suggests the necessity of more thorough investigation into the influence of race upon the production of disease. The census reports show fifty cases of mutism among colored people to sixty-six cases among white people. Is this difference due to race influence, or to the neglect or inability of the negro to receive observation and attention? The only reports in reference to causation of mutism among the negroes, show a nearly equal proportion of mutism from cerebro-spinal fever with the white race, and the same is true of all kinds of alleged causes. Professor Murrell's objection to including under the term "colored" all possessing acknowledged African blood, is undoubtedly good, but even his extensive experience among the so-called "colored" people cannot have brought to his attention any very great number of those of uncontaminated African blood.

Dr. R. Tilley, Chicago, Illinois: I wish to express my high appreciation of the paper which has just been read, and I am sorry the author is not present to receive my compliments. The subject is so completely new to us who practice in the North, that it is necessarily difficult to speak upon it. The subject is, however, peculiarly interesting to me, in connection with the relative susceptibility of the negro to the ravages of syphilis. I refer to this subject in a paper, which I hope to have the honor of presenting to the section. The statistics presented to us are exceedingly interesting, but require further corroboration, and it would be important, if we could bring any influence to bear upon the officers of the next census, to render its reports more exact on this question. The writer's theoretical explanation of the absence of impacted cerumen among the negroes, because of the large size of the external meatus, scarcely corresponds with my observation. For, although I have had very little experience with the negro, yet there is a marked difference in the size of individual canals, and it has been my observation that large canals are more associated with impacted cerumen than the smaller ones.

Dr. L. Turnbull, of Philadelphia, Pennsylvania, remarked that, if he understood the paper, no mention had been made of fibrous tumors of the lobe of the ear in the negro. In his manual Diseases of the Ear, published in 1872, he had made special mention of the great tendency of the negro to this form of disease. He had seen several cases among negro girls of one, two and in one instance three such tumors, which he removed by operation. In his clinic, he has, every year, several cases in the negro and mixed races, of acute otitis media; by negro he meant, not a brown or yellow, but a *black* man.

Dr. S. O. Richey, Washington, D.C. Among charity patients of this country are to be found many Irish. Those of them over forty years of age who have ear troubles, commonly have very large meati; often impacted cerumen, and persistently impaired hearing, associated together. No one of these symptoms is the cause of any other, but we have to look to a more central common cause in the nervous system. The torpid mental and nervous organization of the negro stands in strong contrast to his exaggerated emotional nature, the offspring of his ignorance and superstition. But, in this torpor of his nervous system, we may find some explanation of his comparative freedom from progressive deafness. Keloid of the lobe of the ear is not uncommon in the negro race.

Professor G. E. Frothingham, Ann Arbor, Michigan, said that, though Professor Murrell's paper had already been quite thoroughly and ably discussed, he wished to call attention to the statements it contained, that were important in connection with the report made yesterday by Dr. Bishop, of Chicago. He desired to do so, because they tend to support the theory which he advocated in the discussion of Dr. Bishop's paper. Professor Murrell declares that middle-ear inflammations seldom occur among the colored people, and that they nearly all live in dwellings thoroughly ventilated, not from architectural design but from incomplete construction. They were thus freed from exposure to contaminated atmosphere, which has a tendency, through the germs which it contains, to produce naso-pharyngeal catarrhs and acute and chronic inflammations of the middle ear, especially when the changes of temperature are sudden and extreme. The conditions under which the population lives, from which Dr. Bishop had gathered his statistics, and, so far as inflammatory diseases of the naso-pharyngeal and tympanic cavities are concerned, the results are just the reverse; Dr. Bishop finding these inflammations prevailing to a great extent; Professor Murrell finding them very infrequent. Professor Frothingham believes the infrequency of impacted cerumen can best be explained by the absence of chronic aural catarrh. He has found this to exist in a large proportion of the cases of impacted cerumen, to such an extent as to impair the hearing seriously, often leading to progressive deafness. In many of these cases, the ceruminous glands are stimulated, and, as in many other cases of over stimulation of glands, we get an excessive and pathological secretion. This, with the desquamated epithelium, from the same cause, also in excess, leads to impaction. He is more inclined to this view than the one offered by Dr. Richey.

Dr. H. B. Young, Burlington, Iowa, agrees with Dr. Tilley in the idea, that large straight canals do not, *per se*, tend to prevent accumulations of cerumen. Experience shows the large and straight canals quite commonly affected in this way. It has also been noticed that in some cases there appears to be an hereditary tendency. If parents have it, adult sons and daughters may expect it.

Professor John F. Fulton, of St. Paul, Minnesota, followed with a paper on

PRIMARY INFLAMMATORY DISEASE OF THE MASTOID ANTRUM.

Primary disease of the mastoid cells and antrum is sufficiently common to merit much careful study and classification. Although much of the disease that attacks this region is secondary to inflammation extending from the tympanic cavity or external canal, there can be no doubt that the reverse of this is also true. Acute primary inflammation, either suppurative or non-suppurative, of the pneumatic spaces is extremely difficult to diagnose. The symptoms are obscure and are often referred to parts remote from the seat of disease, therefore a careful clinical history is most important.

The author has known this trouble to be diagnosed as neuralgia, and the patients allowed to suffer for weeks and months, until the trouble has been developed and relieved by spontaneous opening of the cavity's wall. Some of the suppurative cases result in a sub-periosteal abscess behind the auricle In the present state of our knowledge a positive diagnosis of primary disease of the mastoid cells cannot always be made, as neither the symptoms nor etiology have been sufficiently studied. Pain is the symptom most relied on, but this is sometimes very deceptive, as it frequently is referred to regions of the skull quite distant from the seat of the trouble; it is, however, apt to be constant, "stinging," throbbing, and tearing. The patient gets no rest, the discomfort being much increased at night. A diagnosis is best arrived at by a process of exclusion.

There is no trouble with any other portion of the ear; no dullness of hearing, no tinnitus. The diagnosis is rendered almost certain if a swelling develops itself behind the ear *late* in the attack. Disease of the mastoid cells runs rather a slow course. Those of strumous or syphilitic origin are very slow. As soon as the diagnosis is made the mastoid should be opened, when relief will be almost instantaneous.

Professor E. DeRossi, Rome, Italy, said that pain cannot be regarded as a pathognomonic symptom of primary inflammation of the mastoid. He said that on that one symptom alone he would never decide to perform an operation such as trephining the mastoid process. He spoke of a case of primary epithelioma of the mastoid, of which the principal symptom was pain. Tuberculosis of the temporal bone is also accompanied by persistent pain.

Dr. J. Baratoux, of Paris, France, said: I believe that the mere symptom "pain" is insufficient to allow the giving of the diagnosis of inflammation of the mastoid apophysis. I would not be guided by this mere symptom to decide upon so serious a surgical operation as trephining of the mastoid apophysis, inasmuch as it is extremely rare that we do not meet some other symptom than that of pain. There exist, in fact, in these cases other signs of the middle-ear disease which attract the practitioner's attention.

Dr. S. O. Richey, Washington, D. C., believes pain alone, or even in case of chronic suppuration of the middle ear, is an insufficient indication for perforation of the mastoid process, in all cases. He gave a brief history of a case of chronic suppuration, of eighteen years' duration, in which there was absence of the drum-membrane, and of the ossicula, except the stapes. There was also persistent pain, extending from the mastoid region of that side, over the occiput to the vertex. The patient twice in two months fell, unconscious, the fall each time being followed by an escape of dark, fetid pus from the ear. He recovered with a cicatricial drum-membrane, without trephination. Such cases make a surgeon doubtful when he should trephine.

Dr. H. B. Young, Burlington, Iowa, said, concerning the unreliability of pain alone, as an indication for perforating the mastoid, his experience with at least one case will encourage conservatism. This case had the characteristic mastoid pain, without external signs of inflammation. Perforation was suggested, and Knapp's position explained. It was acceded to, but before it was done the mouth was examined, and carious teeth found. The perforation was postponed, the teeth were extracted, and eventually, though slowly, the pain subsided.

Professor G. E. Frothingham, of Ann Arbor, Michigan, then read a paper entitled

THE INDICATIONS FOR THE ARTIFICIAL PERFORATION OF THE MASTOID PROCESS, AND THE BEST METHOD OF PERFORMING THE OPERATION.

From its tendency to involve the brain and thus endanger or destroy life, inflammation of the tympano-mastoid cavity is, perhaps, the most important of aural affections, and one that, in spite of the progress which has been made in otology within the last twenty years, has many mooted points that must be settled before we can diagnose them with sufficient accuracy, or lay down positive rules for treatment. Among these questions, "What are the indications for the artificial perforation of the mastoid process?" and "What is the best method of performing the operation?" are, perhaps, the most practical. They stare not only the specialist, but the general practitioner, in the face, and upon their correct answers the life of the patient will often depend. It is true we have much more precise knowledge on these subjects than we had even a dozen years ago, but our knowledge is still so vague, and our rules for action still so indefinite, that the most accomplishd aurist will often be undecided how to act. He may regret, perhaps, after the autopsy that he did not perforate as thereby he might have saved his patient. On the other hand, he may regret that he did, or that he did not operate by a different method. To a certain extent this uncertainty is due to the inherent difficulties of the subject, and will, perhaps, always exist; but to a large extent it will probably be removed by accumulated experience and wisdom, and further records of cases are desirable as contributing to this result. Some of this vagueness may be eliminated, I believe, by a more extended discussion and analysis of facts already recorded, and it is in this hope that I present this paper for the consideration of the section.

A brief consideration of the anatomy of the mastoid, and its more important anatomical relations, and also some of its more important abnormalities in form or structure, or in the relation of those surrounding parts that affect operative procedure, will be essential to the discussion of the more practical questions found as the heading.

The internal surface of the mastoid bone is smooth, and presents as one of its prominent features, a deep curved groove, known as the sigmoid fossa, in which is lodged a portion of the lateral sinus. In this fossa is generally found an opening for the vein transmitted to the lateral sinus through the mastoid foramen. The situation of the sigmoid fossa with reference to the mastoid process, and posterior wall of the external *meatus auditorius* is of great importance, and, until recent years its variations were undetermined and little regarded. According to Hartmann's measurements, which agree with those of Bezold and others, the distance between the posterior wall of the meatus and the sigmoid fossa is often only a few millimeters. A sharp forward curve of the transverse sinus toward the wall of the auditory canal is very common. In one hundred temporal bones measured by Hartmann it was but five millimeters in one case. In five cases it was only six millimeters. In six cases it was only seven millimeters, while in forty-one of the cases it was one centimeter or less. The maximum was nineteen millimeters, while the average was only eleven and a half millimeters. In the operation of opening the mastoid process the danger of wounding the transverse sinus will be greater in proportion as this distance is smaller, and as no operator can tell when he may chance upon one of these cases, he should avoid those methods of operating which do not allow of ready inspection of the wound as he proceeds.

The floor of the middle cranial fossa has an important relation with the superior mastoid cells that must be contantly borne in mind while opening into them. The floor of this fossa lies but a little above the external auditory canal, being separated from it by only a thin lamella of bone. The course of the facial nerve in the *canalis facialis* turns sharply backward on reaching the inner wall of the tympanum, and, in its course from above the oval window downward and backward to the stylo-mastoid foramen, it lies quite near the upper and deeper mastoid cells, and is in danger if these are perforated carelessly or too deeply. It may be reached in some cases at a depth of five-eighths of an inch. In penetrating with a drill some observe the rule to go no deeper than this. In using the chisel, as the wound is open for inspection, this structure may be avoided. The posterior and horizontal semicircular canals have also such relations to the mastoid cells, that in penetrating with the drill at a depth of more than five-eighths of an inch they are liable to be encountered, especially if the drill is directed too far forward. In operating with the chisel they may be more surely avoided. The occipital artery is lodged in a deep groove at the lower and inner side of the process. The apex of the process serves for the attachment of the sterno-mastoid, splenius capitis, and trachelo-mastoid muscles. The digastric muscle is inserted into a deep fossa, situated on the inner and posterior surface. A mastoid abscess occasionally makes an opening for itself at this fossa, where the outer wall is often thin, or at the apex of the process, and burrows under these muscles deep into the neck. I have met with two such cases in my practice.

The size of the whole process varies much in different individuals, generally being larger in those who are strong and muscular, and in males than in females. The number and size of the mastoid cells vary greatly in different individuals, and even in the same individual on the two sides. They are separated from each other by thin perforated walls of bone, and generally communicate quite freely with each other and through the *antrum* with the tympanic cavity. In some cases a single large space is found in the center of the bone and the cells exist in the form of diplœ in the walls of this central space. In the child, the wall separating this cavity or central space from the lateral sinus is quite thick relatively to the outer wall, and hence meningitis or cerebral abscess as a result of inflammation of the mastoid is comparatively rare. The outer wall is, also, so thin, in childhood, that it is often penetrated with the knife on making Wilde's incision, and is easily opened with a blunt probe or gouge, in cases requiring it.

Although it is now twenty-six years since the operation of perforating the mastoid process was revived and established as a necessary operation under certain circumstances, the indications which call for the operation are still imperfectly formulated, and even the evidences of retained pus are so imperfectly understood that the most experienced operators have frequently been mistaken in their diagnosis, and disappointed by finding no pus where they had confidently expected and thoroughly sought for it. It is hardly sufficient consolation under such circumstances to find, as often happens as an outcome, that the operation has done good by the influence of the traumatism upon the diseased bone, but until very many more cases are reported and studied such disappointments will continue frequent, and the operators will be obliged to explain the mistakes, and justify the operations as best they can. Indeed, the probabilities are that a considerable element of uncertainty will always exist as to the presence or absence of pus, or as to the exact condition of the interior of the bone, even though the indication is plain for perforation.

There are many cases, however, in which little doubt need exist. In these cases there is severe pain in the mastoid, increased perhaps at night, and shooting in every direction, and accompanied by tenderness and swelling over the bone, with elevation and forward displacement of the auricle. The patient has a weak and rapid pulse, and anxious, haggard countenance, dry and coated tongue, and is unable to sleep or take sufficient food. There is a failure of mental and muscular power, irregular chills and fever, while every effort at moving, especially every jar, causes increased pain in the region of the mastoid. Such symptoms point most positively to the nature of the affection and constitute a positive and urgent indication for immediate perforation. Yet such cases, not a generation ago, were generally misunderstood, disregarded, or vaguely diagnosed as cerebral disease.

In all cases in which acute pain arises deep in the mastoid during the course of suppurative inflammation of the middle ear, and is accompanied by tenderness and swelling over the bone, and leeches and other measures calculated to relieve periosteal inflammation afford no relief, or relief for but a few hours, and on making Wilde's incision a normal or nearly normal condition of the periosteum is found, the external table of the mastoid should be opened, and the opening should not be delayed, but made at once. Even in cases where by reason of the early swelling and tenderness of the soft tissues covering the process, and superficial character of the pain, it seems probable that the disease is mastoid periostitis, if Wilde's incision shows not only change in the periosteum, but the bone to be also changed in appearance, then the operation should be extended and the cells opened into, as the change in the bone may be, and most likely is, due to disease in the mastoid cells instead of the periosteal inflammation, the disease beginning in the bone and extending to the periosteum and other tissues over the process.

With acute symptoms, early operation cannot be too strongly insisted upon, for the danger of delay is great in all cases where pus is contained in the mastoid cells. To operate early is only to follow a general principle that acute abscesses should be opened early, and, considering the close proximity of mastoid abscess to the brain, this rule becomes all the more imperative, especially when we consider the slight danger of an operation and little prospect of spontaneous cure. According to Dr. A. H. Buck's analysis of sixty-seven cases, more than half of which occurred in persons under twenty-five years of age, and two of which occurred in children ten years of age and under, only three made a spontaneous recovery, and all of these were under the age of sixteen years. When we consider that Schwartze attributes but two per centum of the deaths occurring after operation to the operation itself, and that seventy per centum of the cases were cured, and only twenty per centum died from the disease when operated upon, including those operated on at a late period in the course of the disease, we shall see that conservatism here is an element of danger, and, that when in doubt, it is best to operate. From cases that have come under my observation I should say that it is a general custom to delay operation too long, or to omit it altogether even in cases where it is evidently called for. There is, up to the present time, too much reluctance, even among aurists, to resort to this operation, and it is only eight years since one of our most progressive and justly celebrated otologists, in an extended article on "Acute Purulent Inflammation of the Middle Ear," said: "*Opening of the mastoid process*" was never resorted to in the series of cases under consideration. Of this operation also, I am not a great admirer, and perform it only when the cerebral symptoms are threatening, especially when, at the same time, the secretion has ceased more or less suddenly, the mastoid region red, swollen and tender on pressure, *i.e.*, in cases that show some probability of retention of morbid substances in the cells of the mastoid process."

Schwartze declares that if the skin covering the upper and posterior wall of the external meatus is bulging, and there is retention of fetid pus in the middle ear, with pain in the region, and general fever, the mastoid may often require perforation, even though the external surface is sound and gives no evidence of disease in the cells. In many of these cases the indications cannot be clearly made out, and a considerable degree of uncertainty must exist as to the necessity, or even propriety, of an operation.

When, in the course of a suppuration of the middle ear the temperature rises rapidly, and continues in spite of antipyretic measures, even though there is no marked pain or tenderness in the region of the mastoid, it is warrantable to perforate the mastoid, as there is reason to suspect pus, and pus has several times been found on perforation with this as the only indication for making the operation. In such cases it will always be doubtful whether or not pus is pent up in the mastoid cells, as the symptom of increased fever and general headache may be due either to retained pus, sinus-thrombosis, or brain abscess. I believe the opening should be made, however, as in some cases it may evacuate pus, prevent extension to the brain, and save life. If it should be brain abscess in the vicinity of the affected ear the opening may subsequently be enlarged and allow of evacuation of the abscess. This was done in one case by Truckenbrod, and the life of the patient saved. Even if it is sinus-thrombosis, the suggestion of Zaufal to expose the sinus, ligate it, lay it open and remove the thrombus, is worthy of consideration.

As to the methods of perforating the mastoid process, they have been quite various. When Joseph Toynbee wrote his classical work in 1860, speaking of disease of the mastoid process, he said: "Perforation of the mastoid process also suggests itself, and this operation may doubtless be performed in those cases where the matter is pent up in the cavity of the ear and is causing such urgent and serious symptoms as are likely, if not relieved, to terminate in death. I have never performed this operation, but I should not scruple to do so in a case where the life of the patient was threatened. Considering the large extent of the mastoid cells, it appears to me that the best plan of operating would be to use a trephine over the middle and posterior part of the process and to remove a portion of bone three-quarters of an inch in diameter." At the present time the trephine is probably never used in this operation, but a small trephine about one-fourth of an inch in diameter, was for sometime commonly used in perforating this process. The directions given for the use of the trephine were altogether too indefinite considering the important relations of the part. When Wilde recommended his incision in his book, published in 1853, he did not specify very exactly where it should be situated. If we take his practice as a guide, it should be situated about half an inch back of the attachment of the auricle, a position at first commonly selected in making the primary incision for perforating the mastoid, as will be seen by reference to reported cases.

When Dr. Roosa wrote his extensive and valuable work on "Diseases of the Ear," in 1873, after consulting all the best authorities on the subject, he could give no more definite guide for the use of the trephine in opening the mastoid process, than to direct an incision after the manner of Wilde, parallel with the attachment of the auricle, and that after the periosteum had been dissected up, "the trephine should be worked inward, forward, and upward." He says: "There can be no positive directions given as to the depth to which the instrument should go." The operation should go on very slowly, frequent pauses being made to see how far the instrument has gone. It is impossible to say in a given case at what depth we shall reach the cells or free spaces, and thus make an outlet for the pus. Dr. Agnew was obliged to go five-eighths of an inch in one of his cases and then only found sclerosed bone. Dr. D. C. Ambrose, removed a piece one inch long from the mastoid process of a young woman of twenty-one years of age. The cell structure will ordinarily be found at a depth of from one-sixth to one-fourth of an inch."

We can hardly wonder that with such directions there was a hesitancy on the part of physicians to attempt this operation.

The employment of drills soon succeeded the use of the trephine in opening the mastoid cells. Those known as Buck's drills, because recommended by him, were most used, and are generally recommended even in the latest text-books by American authors. These drills are entered usually about a quarter of an inch behind the external auditory meatus, and a little below the level .with the upper wall of the canal, and by pressure and a rotatory motion, caused to penetrate the bone in a direction upward, forward and inward.

The opening thus made is usually enlarged by the use of a bit, conical in form, which grinds through the external surface, enlarging the opening, it is true, but by bruising the bone and leaving a ragged surface, which is incapable of perfect cleansing and liable to lead to absorption of poison and consequent constitutional disturbance. Owing to the pressure that must be exercised in order to penetrate solid bone with reasonable quickness, the drill may be driven suddenly inward as the cell structure is reached and resistance ceases, and do fatal injury to the lateral sinus or membranes of the brain. No ordinary precaution, such as stop arrangements, extension of a finger by the side of the drill while at work, etc., etc., which are usually adopted in operating, will wholly guard against this, and in the excitement of an operation these precautions are sometime wholly forgotten. Arthur Hartmann tells us that when he operated on the cadaver with these drills after the manner recommended by Buck, who sets the drill a little below the upper border of the external canal and penetrates inward and a little upward and forward, he penetrated the middle cranial fossa with the drill in three cases out of one hundred. If this happened in operating upon the cadaver, with nothing to disturb the operator, how much more frequently might it happen to an operator disturbed by the responsibility of an operation upon a patient who is, perhaps, acting badly under an anæsthetic, and where in seeking pus he often penetrates to the greatest depth sanctioned by the authorities? When we consider, also, the variable position of the lateral sinus, as determined by Hartmann and others, we shall not wonder that Schwartze characterizes this mode of operating as a "drill into the darkness."

The use of a dental engine to drive the drill, by its rapid rotation allows the operator to penetrate the bone with very slight pressure and to determine when the cell structure or a pus cavity is reached without so much danger of a sudden plunge of the drill as when using ordinary hand drills, but it is still a "drill into the darkness," and the opening made needs further enlargement, which is usually made by a burr which bruises the bone and leads to dangers already mentioned. It may be said that the sharp gouge or chisel may be used to enlarge the opening instead of the burr, but in that case there is no need of a previous use of the drill, attended, as it is, with these dangers.

We are told by Von Troltsch that the first operation of this kind ever made was performed by Jean Louis Petit by means of a gouge and hammer. In modern times we have not only seen the operation itself firmly established, but Professor Schwartze has also plainly demonstrated that the method employed by the great French surgeon is the best and safest that has ever been devised.

The chisel should be applied on a level with the upper wall of the meatus, at first at an angle of about 45°, and the bone chipped off in thin layers as close to the posterior wall of the meatus as possible, and extending downward well toward the apex of the process. In working the chisel either pressure by the hand, or light blows from a small wooden hammer may be used. Five-eighths of an inch is the limit as to depth according to many authors, and beyond that we are in danger of injuring the facial nerve, semi-circular canals, or other important structures. Antiseptic precautions should be observed both during the operation and the after treatment. The opening should be kept patent by means of a small silver or rubber drainage tube, and the most careful attention bestowed in every respect, as the after-treatment often requires the exercise of even more knowledge and skill than the operation itself.

Even in the cases where it is regarded as applicable, the drill leaves too small an opening, very liable to close early by granulations or cheesy pus, and thus lead to a recurrence of the symptoms, or necessitate another operation to remove the obstructions. The opening made by the drill, even when made by skillful operators may, from its necessarily limited extent, fail to reach the pus, and patients so operated upon have died with symptoms that caused the operator to think that pus existed at the time the operation was made, but was retained in a portion of the process not penetrated. In chiseling, we generally lay open more of the cells, and if we wish, can lay them open extensively, while with the drill we open into the antrum, or but few cells at most. The other cells may not freely communicate with the cells opened into by the drill, and thus retention of pus is more likely to occur than after the extensive opening of the cells and thinning of the external table of the mastoid that results in the perforation with the chisel.

Another objection of great importance is the nature of the wound made by the drill. The blood-vessels and lymphatics of the bone are bruised and injured in such a way that it becomes difficult to cleanse their extremities from septic material, which is more likely to be absorbed and lead to severe constitutional symptoms, than when the wound is made with a sharp, cutting instrument, leaving a smooth surface which is easily cleansed and kept free from poisonous germs.

The method of opening the mastoid process by the chisel is adapted to all classes of cases. It is easier of execution than perforation with the drill, and according to my experience does not require, for its execution, "more time and more manual skill," as stated by Dr. Buck.

I believe the superiority of Schwartze's method is sufficiently well established, and its advantages are of so great importance that it should be generally urged for adoption until some still more improved method may be devised.

Professor E. De Rossi, Rome, Italy, asks if Professor Frothingham has ever practiced opening of the mastoid process in chronic suppurative inflammation of the middle ear. It should be noted that Professor Frothingham is among the first to give so specific directions about the method of opening into the mastoid cells.

Notwithstanding the high authority for it, he has not been accustomed to perform the operation in cases of simple chronic suppurative inflammation.

Dr. J. Baratoux, Paris, France, said: I fully agree with the opinion expressed by Professor De Rossi, relative to surgical interference in affections of the mastoid process. I believe that we are too often tempted to perforate this process for affections which can be efficiently relieved by other means.

In actual practice I rarely have to resort to this operation. I almost always resort to other means such as early ice applications, or Wilde's incision; even in those cases which exhibited fever, delirium and pain. I have often made Wilde's incision when I have felt sure that I should find no pus beneath the periosteum, but experience has convinced me that such an incision often suffices if the precaution is taken to give a free exit to the excretions from the external auditory canal. In many cases when, according to my observation, other surgeons would have opened the mastoid cells, I have succeeded in affording permanent relief by the use of insufflations, perforating or enlarging old perforations of the membrane, antiseptic dressing, and Wilde's incision. So far these simple mea ures have given me such satisfactory results that I do not hesitate to say that cases demanding perforation of the mastoid cells are rare. I say this, conscious of the fact that many surgeons do not hesitate to perform the operation, even in comparatively insignificant affections of the middle ear.

My observations are based on an experience similar to that of my confrerès, and I have never yet been obliged to perform perforation of the mastoid cells when, according to my judgment Wilde's incision and the other precautions I have referred to have been deemed at the time sufficient. I consider the necessary opening of the mastoid cells to be rare operations.

Dr. Bishop, Chicago, Illinois: We are much indebted to Professors Fulton and Frothingham for discussing a topic which has not received the attention which its importance merits. Professor Fulton treats not only of primary inflammation of the mastoid antrum proper, but of the mastoid cells, which constitute the whole interior structure of the mastoid process. Had he maintained that the mastoid antrum proper is the seat of a primary circumscribed inflammation, limited to that part only, my experience could not have corroborated his assertion, for I do not remember to have met with a case in which such a diagnosis would have been warranted. Indeed, I cannot think that primary inflammation of the mastoid cells is frequent, except as the result of traumatic injury. I have witnessed the occurrence of the latter, but am not satisfied that it occurs independently of traumatism, or middle-ear disease. I have frequently treated cases which might have been diagnosticated inflammation of the mastoid cells, but which I believe to have been periostitis, in which the inflammation was controlled by cantharidal collodion, or essential oil of mustard, or leeches, combined with anodynes. The pain, redness, swelling and tenderness have often entirely disappeared under this treatment without operative interference, but I have always found such cases either associated with, or following, inflammation of the tympanic cavity.

We should heartily thank Professor Frothingham, for his paper is a most valuable contribution to the history of mastoid disease and its treatment. To be brief, I will give only an outline of my treatment of such cases as require operative procedures. If the abortive measures mentioned fail to arrest the inflammation, and the formation of pus is inevitable, it is my practice to cut down at once upon the bone. If a sinus has formed, it is rimmed out with a drill, and sufficiently enlarged to admit of a thorough examination of the interior of the cavity. All unhealthy tissue is removed; the cavity is treated with scoop and drill, on much the same principle as the cavity of a tooth is prepared by a dentist for a filling; a solution of mercuric bichloride (1-2000) is used to cleanse the cavity, and idoform, or powdered boracic acid, is dusted into and about it. I, like the author of the last paper, use drainage tubes, but the cases in which its use has been omitted have been attended by as fully gratifying results. In closing the wound, the periosteum, which has been preserved is replaced; the sutures are frequent and deeply inserted, and the parts are sealed with antiseptic gauze, absorbent cotton, and net bandage.

My experience is not so extensive as that of some of the authorities quoted, but 85 per centum of my cases recovered completely; the other 15 per centum have passed from my observation before a fair opportunity for a cure, and are classed as doubtful.

I wish to express my dissatisfaction with the use of the trephine in operations on the mastoid where a sinus exists. The sinus does not afford a steady support to the fixation shaft, unless the opening in the bone is very small. If the sinus is large, the superficial layer of bone is likely to be undermined and softened to such a degree that it gives way under the trephine, letting the instrument settle into the cavity suddenly, jeopardizing the dura mater and the lateral sinus. I have never been so unfortunate as to have injured these structures, although I have removed sclerosed bone while the dura mater was exposed; but too much care cannot be exercised to protect these vital structures.

Dr. C. M. Hobby, Iowa City, Iowa, called attention to the hand bone-gauge of the ordinary general operating case, as possessing the requisites, when sharp, of perfect control, rapid work, and of making the best possible wound of bone.

Professor F. C. Hotz, Chicago, Illinois: In regard to the indications for early operation in acute suppurative inflammation of the middle ear, there is one point which cannot be emphasized strongly enough. Surgeons do not see any justification for operating in such cases, as long as the pus has a free outlet through the perforated membrana tympani. I have held and expressed, and I still hold the same views as Professor Frothingham, viz.: that, if judicious treatment continued for several weeks, has proved insufficient to control the suppuration, or, if any medicinal application, even of the mildest character, aggravates the symptoms, and the purulent discharge is very profuse, the mastoid should be opened, there being no doubt that the inflammation has extended beyond the tympanic cavity, and, though the pus has a free outlet though the drum-membrane, the amount of pus in the mastoid cavity is too great to be readily drained off through the perforated drum-head. I have been, quite recently, again impressed with the importance of early operation, by a case of acute suppurative inflammation of the middle ear, which had existed over two months. I saw the case in consultation; there was no tenderness or swelling over the mastoid region; no pain in a distant part of the head; the membrana tympani showed a large central perforation, through which the pus kept welling out, as fast as it could be wiped off. All means had proved inefficient, the discharge being as copious as during the first week. My opinion was that the mastoid was undoubtedly involved, and should have been opened long before. The attending surgeon had not thought of doing so, nor that there was any special indication for the operation, because the discharge of pus was so free and uninterrupted, through the drum-head. The operation was not done, and a week later the patient died of acute meningitis.

Dr. G. W. Allyn, Pittsburgh, Pennsylvania, wished to know if the writer would include in his indications for opening the mastoid cells, cases of chronic suppuration of the middle ear which had, for years, resisted the usual means of treatment.

Dr. R. Tilley, Chicago, Illinois: When the statement is made, that suppuration of the middle ear demands operation, it is too general; we must distinguish between the kinds of suppuration. If the suppuration persist, regardless of special attention to cleanliness, and the use of antiseptic fluids, and the pus wells up in great abundance, and unquestionably arises from the mastoid region, then the operation may be justifiable. But when the suppuration is from the roof of the tympanic cavity, then the perforation of the mastoid cells would be of no avail, and it is just these cases, namely, a persistent discharge from the upper and anterior part of the middle ear, which has given me, personally, the greatest trouble.

Dr. S. O. Richey, Washington, D. C.: Trephination of the mastoid is like tracheotomy for diphtheria, unadvisable until the last moment, and yet we must be sure not to defer the operation until too late. The Scylla of a needless operation, and the Charybdis of not performing it, or of doing it too late, must both be avoided; and the way in which this is done depends greatly upon the judgment of the individual surgeon. The treachery of chronic suppuration is well known. Often there is no symptom of the approaching fatal termination, until too late for the operation. My mind is not clear as to the unquestioned indications for the operation, and these I would like to hear clearly set forth.

Dr. Blitz, Minneapolis, Minnesota: As far as my experience goes in mastoid disease, I am convinced that the operation could, very often, be avoided, if the disease could be recognized by the practitioner of general medicine, and appropriate treatment be at once instituted, as the family physician sees the patient first, and often treats the patient until the aggravated symptoms and increased suffering frighten both physician and patient into consulting some specialist, who finds the case so far advanced that nothing but perforating the mastoid process will save the patient. Often the patient neglects to attend to a suppurative otitis media; the disease becomes chronic, the tissues, more and more, break down, until exposure to cold, or this gradual extension of the disease affects the mastoid cells. In acute inflammation of the mastoid cells, I have found continued applications of broken ice, in a rubber bag, placed over the ear day and night, together with other remedies indicated by the symptoms, best. In regard to the indications for the operation, I fully agree with the author of the paper, and am glad these points are made so clear. As to the method of operating, I must beg leave to differ with the author. The use of the chisel and hammer seems barbarous, when we can use so nice and efficient an instrument as the drill,—not a small, ordinary one, of perhaps one-eighth inch in diameter,—but a round burr-head drill, of at least one-half inch in diameter; for if the operator has mechanical dexterity he will use the drill to far greater advantage, with more nicety and certainty, and without injury to the parts. If the opening needs to be increased, this can easily be done, and all the rough edges of the wound be made smooth and clean. With our present antiseptic precautions there is no more danger from sepsis after one operation than the other.

The operation is not so dangerous in itself as was formerly believed, and there is no reason to dread it so much, because it certainly cannot do so much harm as the disease, if the operation be delayed, or not done, as many such cases unfortunately might show.

Dr. H. B. Young, referring to Professor Hotz's remarks, said : It occurs to me that no mention was made of a *post-mortem* in your case. Professor Hotz replied that there was no *post-mortem* Dr. Young then reported a fatal case, in which the mastoid was opened *post-mortem* and no pus was found, although it was, from concomitant circumstances, certainly to be expected.

Professor M. F. Coomes, Louisville, Kentucky, said he wished to enter a protest against the use of the chisel in opening the mastoid cells, as he thought the method a rude one, to say the least, and one that ought not to be practiced when other methods are at command. He had opened the mastoid in two cases, in which pus was supposed to exist: in one the cells were healthy; in the other a small quantity of sanious fluid was found. One of the patients improved for a time, after the opening was made, was able to be on his feet for fifteen days, and finally died in convulsions. No *post-mortem* was permitted. The other patient died in a comatose condition a few days after trephining. The autopsy showed that the pus passed from the middle to the internal ear, thence through the bony wall into the cranial cavity. The auditory nerve in the canal was in a state of high inflammatory action.

Dr. Toeplitz, New York, N. Y., said in his practice opening of the mastoid has never been made without a distinct indication, *i. e.*, tenderness, pain, swelling, localized headaches, increased temperature, etc. Chronic suppurative otitis, with only a suspicion of pus in the mastoid cells, is not considered a sufficient indication. The operation was always performed with the chisel, which has proved satisfactory in every respect. Are we justified in performing the operation in cases where the brain and the meninges have been involved? I had occasion to make two observations as to this point:

(1) A patient had suffered from chronic suppurative otitis five years previously to the last affection. When I saw him first I diagnosticated sinus thrombosis, and although I had for him no hopes, the operation was performed. The patient died four days after. The autopsy confirmed the diagnosis. (2) Three and a half months after opening the mastoid, the patient showed distinct brain symptoms; intense headache and paralysis of the external rectus of the left eye. I opened the mastoid process, which had completely closed, whereupon the symptoms subsided. Cure, after two months.

Dr. W. F. Holcombe, New York, N. Y.: I am firmly convinced that the drill used by dentists is the best which can be used for opening bony parts for the exit of pus. These drills are of many sizes. Great care must be used in not pushing the drill too hard, as it cuts more rapidly than the hand-drill.

Dr. Paddock, New York, N. Y.: Found the drill run by the dental engine valuable for drilling into the mastoid cells, when the operator preferred the drills to the gouge, as it is perfectly under the control of the hand of the operator, and, with a small drill, any size of opening can be cut. The small amount of pressure required makes it useful.

Professor Frothingham, in closing the discussion of his paper, said he would be very brief, as the subject had been already very fully debated. In answer to Professor De Rossi: he had not been in the habit of perforating the mastoid for simple chronic suppuration of the middle ear, and he advised it only when an examination and study of the case revealed a greater amount of secretion than would be ordinarily furnished by the tympanic cavity alone, with a further probability that it was poured into the tympanic cavity from the mastoid cells, and not from some other direction. This is a matter for careful study and consideration, and from the result of this study of the case, we should determine whether or not benefit would be likely to follow the operation. He had no doubt many obstinate cases of chronic suppuration of the middle ear could thus be cured. He would further remark that the situation of the mastoid cells and antrum, with referance to their communication with the tympanic cavity, is such as to lead usually to a retention of pus in the mastoid in most cases where the cells had become extensively involved in the suppurative process. In order to drain this cavity he believes an external opening preferable, more safe than any that can be made through the wall of the meatus, or tympanic cavity. The external table of the mastoid in such cases should not only be opened, but the opening should be large and extend well down toward the apex of the process; indeed, the temporal bone, which he had exhibited to the section, showed a distance between the posterior wall of the external meatus and the sigmoid fossa of only four and six-tenths millimeters; it showed, also, the necessity of making the perforation so low down that the cells should not be reached above the central axis of the auditory canal. An objection had been made that surgeons dread to do the operation, and that it should be resorted to only when the indications are plain. In his paper he had advocated a more frequent resort to the operation than he had been accustomed to practice. In reviewing his past experience he did not doubt that he could have safely relieved many patients by an operation—have effected a more speedy cure than nature had done without this aid. Fortunately, he had had no case of death where operation had thus been omitted in his private practice, but several cases had come to his clinic at a stage of the disease beyond any hope, in which an early operation might have saved life.

He did not wonder that surgeons dreaded the operation with drills. While engaged in general surgery, he had performed nearly all the capital operations, and had approached no operation, not even opening the sac of an aneurism of the common carotid, with more solicitude than some of these operations upon the mastoid with a drill, which he regarded, with Schwartze, as a "drill into the darkness." Who, with such a method of operating, in such an important and variable anatomical region, can feel that he is not placing his patient in great danger from the operative procedure alone. The object of treatment is to *lessen* the dangers to which the patient is exposed, and not to increase them. With only unsafe methods of operating, we may rightly withhold surgical interference, when with available safer methods we should urge it. After careful study of Schwartze's method, he is convinced that it is attended with so little danger and difficulty that we may urge it in many cases which formerly would have been left to the expectant plan. He believes that the cases of spontaneous cure reported could have been more speedily cured by perforation and with less danger. The subject is too extensive for exhaustive consideration at any single conference, but that the mastoid is as liable to primary inflammation as other bony structures, seems to be a fact overlooked, or but imperfectly considered by the profession. Professor De Rossi had very truly said that pain, alone, is not generally regarded as sufficient indication for the operation. The pain that calls for perforation of the mastoid must be studied. It is generally a deep-seated, throbbing pain, more severe at night, and resists all other efforts for its relief. The patient is unable to attend to any duty. We often find it affecting other bones, without external evidence of disease. He has seen many cases in which the tibia was affected. His esteemed colleague, the late Professor A. B. Crosby, had taught him how, speedily, to cure many of these cases, by boring into the bone with a common gimlet, and he opened into the mastoid in this way. An opening into the bone affords most speedy relief in inflammation of the bone. He has seen operations of the kind, with immediate and permanent relief, though no pus was found, and no appearance of sclerosis was visible to the eye. There are numerous cases in which perforation of the mastoid in search of pus has led to relief without finding pus or sclerosis. He believes pain, alone, under some circumstances, constitutes a positive indication for perforation.

---

### SECTION ON MILITARY AND NAVAL SURGERY AND MEDICINE.

SECOND DAY—AFTERNOON SESSION.

The discussion of abdominal surgery was first taken up.

Dr. W. N. Hingston, of Montreal, Canada, thought that when any doubt existed as to the course of the bullet, operative procedures were necessary. The speaker considered the paper by Professor Senn the most able he had ever heard.

Regarding Dr. Homans' paper, he mentioned that ventral hernia occurred in ten per centum of his cases ; this the speaker considered rather large. Dr. Homans had stated he had operated for nervous affections in five cases; one was relieved, the other four were not; other professional men had given the opposite result. The speaker remarked that he believed Dr. Homans, but did not believe the others.

Dr. J. B. Murphy, of Chicago, Illinois, remarked that in regard to the incision being made in the median line, he was in one case compelled to deviate from this rule in order to reach the gut. If the operation is not dangerous to life, he considered it compulsory, and if the bowel is not injured it does no harm.

Professor W. F. Peck, of Davenport, Iowa, remarked that as regards foreign substances in the peritoneal cavity, he had put in forty-seven sutures in an ovarian cyst.

Dr. S. C. Gordon, of Portland, Maine, stated that out of eighty cases he had seen three of ventral hernia; he had operated in thirty-three hysterical cases, and twenty-five would say today they were cured, which differed from other statements made.

Dr. Cowden stated that he himself suffered from invagination of the gut (referring to Dr. Senn's paper), but the finger inserted into the bowels could just reach it; after days of suffering a long rubber tube was inserted into the rectum, and a large bottle of water secured at the other end and gradually raised; suddenly there was a sharp report and the abdomen was distended. The speaker thought the gut had been ruptured, but the invagination had been reduced, Professor Senn's proofs upon dogs notwithstanding.

Professor T. G. Richardson, of New Orleans, Louisiana, remarked that his experience in these cases was that the percentage in the negro was in excess of that in the white man.

Professor Donald MacLean, of Detroit, Michigan, read a paper on

THREE CASES OF SURGICAL DISEASE OF THE KIDNEY WITH AN ACCOUNT OF OPERATIONS PERFORMED FOR THEIR RELIEF, THE COMPLICATIONS WHICH AROSE, AND THE RESULTS.

The first case mentioned was that of a woman, twenty-one years of age, who was supposed to be suffering from an ovarian tumor. Laparotomy was performed. The patient entered the hospital October, 1884, and in January, 1885, was perfectly well.

The second case was a woman, forty years of age, the same result being secured ; all trace of kidney-tissue had disappeared in the immense cyst.

The third case was a child, twenty-two months of age; this case resulted fatally.

The next paper, by Sir Thomas Longmore, of Netley, England, relative to

THE DESIRABILITY OF PROVIDING SOLDIERS IN SERVICE IN THE FIELD WITH SOME FORM OF PRELIMINARY DRESSING FOR WOUNDS, was read in abstract by Dr. Marston, of the British War Office : The custom of issuing " a first field dressing to soldiers in warfare," was first introduced in the British army, by general order, during the Crimean war, in the year 1854. Since that date they have been more or less used by the Continental armies, and they now form one item of the authorized field equipment of soldiers of the German army. The paper discussed the question of the materials most suitable for the purpose, and quoted the opinions of eminent military surgeons concerning their use. The paper was accompanied by a dressing designed by the author for this purpose. It possesses the advantage of being both complete and compact.

A translation in abstract, of a paper by Professor Von Esmarch, of Kiel, Germany, upon the same subject, was read by Dr. M. J. Stern, of Philadelphia, Pennsylvania. The author described the requisite qualities of a dressing for the purpose, and advocated the use of antiseptic materials for its composition. A sample, designed by the author in accordance with his views, was exhibited.

In the discussion which followed, Dr. Reed Brockway Bontecou, of Troy, New York, strongly advocated the use of a preliminary dressing, and showed that even large wounds received on the battle-field could be to some extent freed from complications, if at once occluded by an antiseptic dressing. But the difficulty with all the dressings heretofore proposed for army use is that they cannot, in all cases, be applied by the soldier himself; in a wound of the shoulder, for instance, the soldier would find it impossible to apply, or at least to keep such a dressing in position. The speaker presented a dressing designed by himself to overcome this difficulty, composed of a square piece of adhesive material, to the center of which is sewed the occlusive portion of the dressing, which, being placed over the wound, will be kept *in situ* by the adhesion of its border to the skin. Before applying it, he advised, in cases of gun-shot wounds, to thrust into the wound of entrance a pledget of antiseptic cotton; the occlusive pad of the dressing, just described, is composed of this material, within which is included a mixture of salicylic acid and iodoform.

Dr. Joseph K. Smith, U. S. army, thought that if a primary dressing is used, it should be strictly antiseptic, but he deemed it necessary that every soldier should carry such a dressing.

Dr. Porter, U. S. army, related his personal experience in proof of the value of a primary antiseptic dressing. He had been accidently shot while hunting in the Adirondacks, many miles from any settlement. The wound was packed with iodoform and gauze, and this primary dressing was not removed for some days, in fact, it was never entirely removed. He was throughout comparatively free from pain, his wound healed rapidly without suppuration, and within a month was well.

The next paper, by Dr. Neuderfer, of the Austrian army, entitled

ON THE PRESENT STANDPOINT OF ANTISEPSIS AND THE BEST MODE OF ITS APPLICATION IN WAR, was read by title, after which a paper, by Dr. B. A. Watson, of Jersey City, New Jersey, on

THE PRIMARY TREATMENT OF GUNSHOT WOUNDS, was read. The author first adverted to the requisite qualifications of a military surgeon, and the duties required of him; discussed the selection of a proper site for a field station, and the means which should be adopted primarily for the arrest of hæmorrhage. As the ligation of arteries is only, under the circumstances, exceptionally required, compression should be relied on to control hæmorrhage. The author urged the importance of removing all foreign bodies from the wounds, and advocated the occlusion of the wounds of entrance and exit with aseptic bandages and compresses. Thoracic and abdominal wounds cannot be treated at the field station, and should be removed from the scene of action as rapidly as possible. Bayonet wounds should be treated in the manner suggested for gunshot injuries, while wounds produced by large projectiles, fragments of shells, and the like, should receive the treatment usually recommended for injuries caused by machinery and railroad accidents. The author closed with a few directions for retaining fractured bones in apposition, and the means employed therefor.

The discussion of the paper was opened by Dr. Cullen, who averred that it was impossible to comply, upon the battlefield, with all the condiions imposed of strict antisepsis; the speaker spoke of his large experience during the late war, and characterized all attempts of this nature as utterly futile. Rest, he said, was the best primary treatment, and the blood poured out upon the wound was the best antiseptic. Movement was fatal to many cases; the wounded should therefore be as gently handled as possible under the circumstances, sheltered under trees, and kept in a state of immobility.

Dr. William Varian, of Titusville, Pennsylvania, spoke of the dressing of a wound in its own blood as a procedure taught to the profession many years ago, and while he recognized the fact that many cases did well when treated in this way, he thought it the duty of the surgeon to treat the wound aseptically, in accordance with modern ideas, as it has been proven beyond a doubt, that such treatment insures a larger percentage of successes than by the time-honored procedure lauded by the former speaker.

Dr. G. T. Langridge, of England, agreed with Dr. Cullen so far as the importance of immobility is concerned. The less a wound is interfered with the better the chance of recovery, but a strict antisepsis is a necessity; the speaker had found that antiseptic wool was a better material for occluding the wound in primary dressing than any other. It is extensively used in the British army.

Dr. Carnochan, of New York, N. Y., shared the opinion of Dr. Cullen regarding the primary treatment of wounds in the field, and with him recognized the impracticability of using the antiseptic dressing on the field of battle, although he is in favor of it in private and hospital practice, where there can be no doubt of its great utility. The soldier himself becomes too indifferent, by reason of constant exposure to danger, to give heed to such details and appliances as the antiseptic treatment demands, so that any dressing designed to be applied by the soldier himself is without practical value.

Dr L. von Farkas, of Buda-Pesth, Hungary, then read a short paper giving statistics of results of antiseptic treatment of wounds, which were very favorable to the method, and exhibited a form of drainage-tube and a speculum designed by himself for the exploration of gunshot wounds.

The next paper was by Dr. Eli A. Wood, of Pittsburg, Pennsylvania, entitled

THE IMPORTANCE OF THE GOVERNMENT'S SECURING AND PRESERVING VITAL STATISTICS IN THE ARMY AND NAVY FOR THE BENEFIT OF SUBSEQUENT APPLICANTS FOR PENSIONS.

He emphasized the duty of the Government towards its soldiers and seamen, and alluded to the cost, trouble, and injustice to applicants for pensions where no complete and authentic records are preserved. He urged that the Pension Office should be so administered that not only individual interests should not suffer, but also so as to protect the Government against imposters. He urged that all military and naval surgeons keep a full record of facts pertaining to the clinical history of all diseases and injuries incident to soldiers and seamen.

The last paper of the day, by Dr. Daniel Smith Lamb, of Washington, D. C., on

THE IMPORTANCE OF INTERNATIONAL REGULATIONS FOR THE MEDICAL TREATMENT OF PRISONERS OF WAR.

They should be treated, not as criminals—harshly treated or robbed of their personal effects—but as human beings worthy of considerate treatment. They should be provided with such food, clothing, shelter, and occupation as will preserve health. If captured while sick or disabled by wounds, or if they become the prey of disease after being captured, they should receive such treatment as will tend to promote recovery. If the captors are unable to afford the proper care or treatment, they should be paroled and returned to their own side.

---

### SECTION ON LARYNGOLOGY.

### Second Day—Morning Session.

Dr. Lennox Browne, of London, England, read a paper entitled,

RECENT VIEWS AS TO THE PATHOLOGY AND TREATMENT OF TUBERCULOSIS OF THE LARYNX.

The tubercular bacilli are generally admitted as a cause of specific laryngitis. Entrance is effected through the air passages, and they are especially liable to accumulate in the upper parts of the lungs, where there is less respiratory action. The disease is usually secondary to pulmonary tuberculosis, and may be due to infection from the bacilli in the sputa coughed up and inoculating some abraded or unhealthy and irritated portion of the larynx, or the germs may find their way thither through the lymphatic system. The bacilli, like all parasites, act as an irritant, and eventually cause breaking down of the tubercular deposits.

They must have an unhealthy or abraded surface on which to locate and thrive, and are prone to choose some weak point. Whether carried by the air, sputa, lymphatics, or general circulation, cases are recorded of infection through wounds and teeth that have been extracted. The author of the paper quoted cases tending to confirm his views.

The state of the health and the assimilation of food together with tissue nutrition, have more to do with the development of tuberculosis than have locality or climatic conditions. Not only may we have laryngeal tuberculosis as a secondary infection, but we find cases where it is primary. The laryngeal symptoms may be the first to attract attention, and even become far advanced before pulmonary lesions can be detected. This is confirmed by many observers. The bacilli are recognized in the sputa, there is pain in the larynx, and difficult deglutition.

The larynx presents specific appearance. We find the infiltration, dwelling, and ulcers, but still can detect no pulmonary lesion. There must have been some neglected chronic laryngitis, or some solution of continuity when exposed to the presence of these germs. Cases are reported where these local symptoms have yielded to treatment before pulmonary complications were added, and the patients were discharged apparently cured.

Treatment.—Where the general system is not broken down, or the disease advanced to the lungs, sea or mountain air and high altitudes, especially in pine regions, are of vital importance. Oxygen, pure air, and the absence of germs are here more to be relied on than the thermal or climatic agents. Dr. Moore has ceased to advise the inhalation of medicated steam; but prefers oxidizing agents. Inhalations of vapor from turpentine, oil of eucalyptus, and menthol he has used with success.

It is well established that tuberculosis is a blood-poison, as is pyæmia, septicæmia, and the like. Germicides should be used, and benefit will result. He places great confidence in atropine, not only as a sedative, but a germicide as well. Arsenic often works in the same manner as does mercury in syphilis. So, also, do the salts of calcium, where there are tubercular deposits. The aniline treatment has not been a success, and the gaseous injections are still on trial. Experiments with sulphuretted hydrogen show temporary improvement, with diminution of the amount of sputa expectorated, less pain and less distress from persistent cough. Still the permanent benefit is doubtful. This treatment needs careful supervision, and should not be trusted to the patients or their friends. The local treatment of tubercular laryngitis gives best results, especially where systemic treatment is also employed to maintain health and nutrition. It is well to use cocaine, and to employ the galvano-cautery or lactic acid to destroy the deposits and induce healthy healing of the parts.

Where the infections are local, success is in proportion to their accessibility.

He does not like iodoform or iodol dissolved in ether, as the ether is too much of an irritant. He prefers a brush made with cotton to the spray, in that it coats the surface better and is pressed into folds which are protected from the spray by the spasm of the larynx. The continued use of the spray he considers to be dangerous to the ciliæ of the epithelial cells. Local and systemic sedatives are emphatically called for. Insufflation is not as good as where emulsions are made with acacia, as they are apt to form cakes. Cocaine gives temporary relief, but morphia, belladonna and balsam are more permanent in their relief of both pain and cough.

The surgical measures are to scrape away the deposits with curette or forceps, under cocaine, and apply lactic acid. To stab or incise may relieve tension and congestion, but is bad in that it gives new foci for infection. For the same reason it is better not to remove glomerules unless respiration is seriously interfered with. Tracheotomy, for the purpose of giving rest to the larynx, is useless or worse. The larynx does not then receive the necessary air and oxygen, and bacilli-infected mucus accumulates. The cold and dry air irritates the lungs and may induce pulmonary complications. Besides, the wound may become infected. Intubation of the larynx likewise causes too much irritation and aggravates the trouble, independent of the risk of blocking up the tube. He refrains from removing an elongated uvula. He does not approve of the recent suggestion of extirpating diseased portions.

Many apparent cures are reported, and he has had such cases himself, but he is inclined to doubt their permanence. One can improve the condition of the larynx, stop pain and cough, cause better assimilation of food, and improve nutrition. There result local relief and apparently satisfactory results, but scarcely a cure, as claimed by Schmidt, Bosworth, and others.

Important suggestions.—(1) Early diagnosis and treatment are important. (2) Do not be carried away with new remedies. (3) Never be too active in destroying tissue ; rather attempt to heal instead. (4) It is better to observe facts and be influenced by the experience of many, than the new ideas of a few.

Dr. Sinclair Coghill, Ventnor, England, advised more effort to alleviate pain and distress which tend to shorten life, and to obtain as much rest for parts as possible, and to look to body nutrition. He also quoted cases of primary tubercular laryngitis from infection. He has seen the greatest benefit from iodoform and morphia by careful insufflation. He has seen cases apparently cured. The medium or tissue in which the bacilli thrive is to him more important than the bacilli themselves. We do not see parasites in vigorous tissues. Those well nourished are not liable to infection. He advises not to rely on local treatment alone, but improve digestion and assimilation.

Professor J. S. Cohen, of Philadelphia, Pennsylvania: Sulphurous treatment improves tissues or soil so that germs cannot flourish as well. He only recalls two cases of recovery from supposed tubercular laryngitis. He has scraped and applied lactic acid, also used morphia, iodoform, and atropia, and he believes in them. He condemns tracheotomy to obtain rest.

Professor E. Fletcher Ingals, of Chicago, Illinois, finds it difficult to choose between high and low altitude. High altitudes are injurious where the disease is advanced. Calcium salts are beneficial; so, also, are the chlorides. The use of sulphuretted hydrogen, in his experience, increased pain and other unpleasant symptoms, so he abandoned it. He also had poor results from lactic acid. Morphia, carbolic acid, and glycerole of tannin with water form excellent wash or spray. Tannin aids in alleviating pain by hardening the tissues. Glycerole of tannin without water is too irritating.

Professor M. F. Coomes, of Louisville, Kentucky, finds that iodoform works best, and uses it extensively for the pain by insufflation after cleansing with Dobell's solution. Whisky and water is excellent to cleanse away the mucus and ease pain so as to allow the patient to eat. He has seen a case recover, even when the epiglottis had sloughed away.

Doctor Devilbiss, of Toledo, Ohio, uses both morphia and iodoform, but with liquid vaseline which is sprayed in. He considers the liquid form of vaseline the very best medium. He uses it with iodine and iodide of potassium.

Professor W. E. Casselberry, of Chicago, Illinois, doubts the good effect of lactic acid as it causes too much irritation, increases the cough, and does harm. The main point is to cleanse thoroughly, and to use morphia and iodoform, or, better yet, iodol, which is lighter and more easily diffused. The temporary effect of cocaine is excellent, but its continued use is bad. It causes reaction with congestion, increased irritation and inflammation. Morphia is better, and with iodoform effect lasts a longer time.

Professor F. O. Stockton, of Chicago, Illinois, has not had any success from scraping ulcers, and uses lactic acid from ten to ninety per cent. He saw a case in Vienna where there was both tubercular and syphilitic ulcerations of the larynx.

Dr. John Mackenzie, of Baltimore, Maryland, is skeptical as to permanent cures, and thinks that they are more likely diphtheritic cases. Has also doubts as to bacilli, with reference to cause and effect. Why will not they be caught in the nose of the same individual when abraded surface exists? He is in favor of constitutional treatment, and of local treatment with bichloride of mercury, 1 to 2,000.

Professor L. Curtis, of Chicago, Illinois, says that atropine is of no use. He sprays the larynx with nitrate of silver, with the result of healing the ulcers.

Dr. Browne closed the discussion. He believes in the bacillary origin of the disease, but treats both bacillus and tissue as well. Note the close relation of bacillus of tuberculosis to that of lupus. One must treat individual cases differently. He has had no good results from large doses of quinine, as it was not assimilated. Iodide of potash proved harmful, by causing weight and interfering with nutrition. It is of benefit only in syphilitic laryngitis, which can readily be mistaken for tubercular.

---

### Afternoon Session.

For special discussion a paper was read, entitled

#### RECURRENT HÆMORRHAGES OF THE UPPER AIR-PASSAGES, BY WILLIAM PORTER, M. D., ST. LOUIS, MISSOURI.

Larnygeal hæmorrhages are immediately dangerous only when beneath the mucous membrane, when they produce a hæmorrhagic œdema and impending asphyxia. Laryngeal hæmorrhage is very easily mistaken for hæmoptysis, but on auscultation one can discover no pulmonary signs. A list of the causes of hæmorrhage from different parts of the upper air-passage was given, with both medicinal and mechanical treatment of each.

Professor Stockton, of Chicago, Illinois, gave the history of a woman who consulted him with reference to coughing up a considerable amount of bright, frothy blood, which apparently came from the lungs. Examination revealed a small pulsating vessel on the right ventricular band, from which there was a slight spurt of blood at each pulsation. After failing to control it with iron, tannin, etc., the vessel was destroyed with electro-cautery.

Dr. Lennox Browne, of London, England, said that it was usually found in connection with syphilitic or tubercular larynx, sometimes caused by intemperance, and may require general treatment as well; straining of the voice may cause a congestion and dilated vessels. In alcoholic cases we frequently find such patches at the base of the tongue, and above the epiglottis.

#### TREATMENT OF LARYNGEAL PAPILLOMATA.

Professor W. E. Casselberry, of Chicago, Illinois, read a paper on this subject.

The treatment resolves itself into the best methods for removal. Forceps, cautery, curette, or a combination. For small tumors forceps are most commonly used, and are best in that they are simplest. For larger tumors they are bad, for the reason that only small portions can be removed at one sitting, and it will have regained its former size before the resulting inflammation will have been sufficiently reduced to admit of another operation. A case was related to illustrate this, where the tumor was afterward extirpated by galvano-cautery. In another case there were a large number of small tumors returning as fast as removed. He had to resort to curette and follow up with cautery. *Sajous'* electrode was exhibited, also a handle with light wires attached to the center instead of the end. He has had excellent results with the cautery. He uses a curette and cauterizes the bases.

Professor J. S. Cohen, of Philadelphia, Pennsylvania, uses sponge for small papillomata, especially when below or upon the vocal chords. He operates through the crico-thyroid membrane for larger ones. He has not found a cautery so successful; it only scorches the mucous membrane, and there is danger of injury to other parts. He believes in crushing with forceps if he cannot remove.

Professor Ingals, of Chicago, Illinois, advocates using chromic acid on a probe and carefully destroying with that agent.

Dr. Lennox Browne, of London, England, for twenty years used sponge, but he now prefers the snare. He gave up sponge because he once lost the end of the applicator in the windpipe and legal complications developed therefrom. He prefers a safe cotton brush with solid and jointless whalebone applicator. For persistent hyperæmia he enjoins continued silence and uses lead coil over the larynx. He drew a diagram showing the situation and size of the papillomata in the throat of the Crown Prince of Germany.

It was nearly the size of a split-pea, and situated on the vocal chord, close to its junction with the arytenoid cartilage, apparently having its base partly in the ventricle. What now worries Dr. Mackenzie is not that it may be malignant, but the persistent laryngeal hyperæmia. His work in Berlin was accomplished with strange instruments, and not well suited for the work. He found that the surgeons there had decided upon removing one-half of the larynx.

Dr. C. Slover Allen, of New York N. Y., gave the history of a case operated on for large papilloma below the vocal chords. It was so large as to almost completely close the trachea. There was great dyspnœa on slight exertion, frequent severe choking spells at night, and extensive loss of weight. As the tumor could not have been forced through the larynx, even if severed, it was removed through a free opening in crico-thyroid membrane. It was taken off by snare, then the base thoroughly extirpated by curette and cauterized. The attachment was just below anterior extremity of left vocal chord. There was no preliminary tracheotomy. The result was complete restoration of voice and health.

Professor Casselberry, in closing the discussion, said that he used twenty-five per centum cocaine, and had no difficulty in controlling the amount of destruction by cautery. He presses the electrode well into tumor before making the circuit. He has not found the cautery fickle; but has had unsatisfactory results from crushing.

Next in order was a paper for discussion on

THE DIAGNOSTIC DIFFERENTIATION OF RECENT TUBERCULOSIS, SPECIFIC AND RHEUMATIC LARYNGEAL DISEASES.

Professor E. L. Shurly, of Detroit, Michigan, gave an abstract of the paper, which can be best shown in tabular form:

| Laryngeal Phthisis. | Syphilis. | Rheumatism. |
|---|---|---|
| | *Hyperæmia or Congestion.* | |
| Occurs later, but not marked. | Always persistent and extended. | Always present not intense; often localized. |
| | *Tumefaction or Infiltration.* | |
| Constant and peculiar; affecting arytenoids. | Not marked. | Rare, except arthritic arytenoid joint. |
| | *Condylomata and Gummata.* | |
| None. | Sometimes. | None. |
| | *Hæmorrhage.* | |
| Rare, as result of erosion. | Very rare. | Common. |
| | *Mobility.* | |
| Slightly affected. | Slightly affected. | Excessive in arthritic. |
| | *Ulcerations.* | |
| Common, develop slowly, scattered, small, irregular, or oval, usually on epiglottis and arytenoids. | May be rapid, but rare; in larynx, solitary; symmetrical and with areola. | None. |
| | *Hoarseness.* | |
| Present. | Slight; also slight dysphonia. | Present; also dysphonia or aphonia. |
| | *Dysphagia.* | |
| Only when epiglottis and arytenoids are affected. | Not marked, unless pharynx involved. | Persistent and constant. |
| | *Pain.* | |
| Absent in larynx. | Absent. | Constant and about neck. |
| | *Expectoration.* | |
| Often contains bacilli. | Mucus. | Slight. |
| | *Cervical Glands.* | |
| Not enlarged. | Enlarged. | Not enlarged. |
| | *Sensation.* | |
| Not perverted. | Not perverted. | Often perverted; paræsthesia; frequent spasm; also chorea. |

CHRONIC RHEUMATIC LARYNGITIS.

Professer E. F. Ingals, Chicago, Illinois, read a paper on the above subject. It has been recognized for several years, but never thoroughly studied. He has found but few cases mentioned in literature; cases are rare, and usually associated with rheumatic diathesis. He has seen cases where the hyoid bone and posterior part of larynx presented the only evidence of the disease. More often it occurs with attacks of articular rheumatism. It is erratic and obstinate. Pain may disappear for a few days and then return. In no case is the pain very severe. Hoarseness and aphonia are marked. Pain is worse in damp or wet weather, but varies from day to day. The larnyx alone may be invaded, or the base of the tongue and hyoid. In some cases it is slightly swollen, in others not. There may be distension and swelling of the arytenoid joint. One may diagnose by excluding neuralgia, phthisis, and syphilis. Often it is confused with neuralgia when no swelling exists. It may last from two months to a year or more, with periods of immunity. Generally there is ultimate recovery, though one case lasted four years.

Treatment.—Cleansing and astringent spray. Salicylate of soda, iodide of potash, alkalies, oil of gaultherium, ext. phytolocca, etc., used with benefit.

Dr. A. B. Thrasher, of Cincinnati, Ohio, read a paper on

RESORCIN IN THE TREATMENT OF NASAL CATARRH.

Resorcin has a great affinity for oxygen, and absorbs it from the tissues; reduces congestion and inflammation by contracting the vessels. In hypertrophic catarrh, it contracts turbinated tissues and effect lasts a long time. The effect of cocaine is only temporary. Resorcin gives good results in infectious skin diseases because it is antiseptic; penetrates skin and kills germs. Cocaine interferes with secretion and the proper functions of mucous membrane. Resorcin gives the same results, but normal functions remain. It is slower in action and more lasting in result than cocaine. Cleanse nose with boracic solution, or Dobell's, and use two to ten per cent. in vaseline or cosmoline; use a spray every second day. For the patient's own use give two to four per cent. It will cure where dilated turbinated tissues exist in from two to six weeks. It allays all redness and congestion. The turgescent state yields better than where there is true hypertrophy.

(*To be concluded in the October number.*)

